# Precision Nutrigenomics in Cultured Finfish: Dietary Regulation of Gene Expression, Microbial Ecology, Metabolism, and Immunity

**DOI:** 10.3390/microorganisms14081786

**Published:** 2026-08-13

**Authors:** Md Hashibur Rahman, Hyuncheol Jeon, Haham Kim, Seunghyung Lee

**Affiliations:** 1 Major of Aquaculture and Applied Life Sciences, Division of Fisheries Life Sciences, Pukyong National University, Busan 48547, Republic of Korea; hashibur@pukyong.ac.kr (M.H.R.); conjp@naver.com (H.J.); haham7@naver.com (H.K.); 2 Interdisciplinary Program of Marine and Fisheries Sciences and Convergent Technology, Pukyong National University, Busan 48513, Republic of Korea; 3 Feeds and Foods Nutrition Research Center, Pukyong National University, Busan 48547, Republic of Korea

**Keywords:** nutrigenomics, aquafeed, fish microbiome, intestinal microbiota, probiotics, microbial metabolites, host–microbiota interaction, transcriptomics, intestinal health, precision nutrition

## Abstract

Precision nutrigenomics requires a diet–microbiome–host perspective because microorganisms can transform feed substrates, generate bioactive metabolites, compete with pathogens, and modify intestinal and systemic gene regulation. This structured narrative review synthesizes representative controlled feeding trials, transcriptomic and targeted gene-expression studies, microbiome analyses, and complementary multi-omic evidence concerning dietary regulations in cultured finfish. The available evidence is concentrated particularly on soybean-derived proteins, lipid-source replacements, selected amino acids and micronutrients, functional additives, probiotics, and fermented ingredients in a limited range of cultured finfish species; therefore, the synthesis is not intended to provide exhaustive coverage of every dietary intervention or finfish taxon. Recurrent host responses involve intestinal inflammation and barrier integrity, nutrient transport, lipid and bile-acid metabolism, long-chain polyunsaturated fatty-acid biosynthesis, targets of rapamycin/insulin-like growth factor (TOR/IGF) signaling, and nuclear factor erythroid 2-related factor 2/Kelch-like ECH-associated protein 1 (Nrf2/Keap1) antioxidant defense. The expanded microorganism-centered synthesis shows that dietary effects depend on microbial niche, substrate availability, community succession, metabolite production, and strain-specific probiotic or pathobiont activity. Lactic-acid bacteria, *Bacillus*-associated interventions, butyrate-generating strategies, fermented ingredients, and microbial biomass may support digestion, immune balance, and disease resistance, but taxonomic shifts alone do not demonstrate functional benefit. Current evidence is limited by extensive reliance on 16S ribosomal RNA (16S rRNA) relative-abundance data, inconsistent digesta-versus-mucosa sampling, inadequate feed and water controls, and weak causal validation. Future precision aquafeed studies should combine host transcriptomics with absolute microbial quantification, shotgun metagenomics, metatranscriptomics, metabolomics, culturomics, histology, and pathogen challenge. Integrating microbial function with host phenotype can improve sustainable feed design, intestinal health, and resilience.

## 1. Introduction

Nutrigenomics is broadly defined as the study of interactions among nutrients, dietary components, genome regulations, and downstream physiological functions [1,2,3,4,5]. In fish nutrition, nutrigenomic approaches are particularly valuable because cultured fish are exposed to formulated diets that differ substantially from natural prey in ingredient composition, nutrient availability, lipid class distribution, antinutritional factors, palatability, bioactive compounds, and microbiota-modulating properties [3,4,6,7,8,9]. Conventional nutrition trials typically rely on growth performance, feed conversion ratio (FCR), feed efficiency, survival, whole-body composition, digestive enzyme activities, and selected plasma or tissue biochemical markers. These endpoints remain indispensable for practical aquafeed evaluation, but they often cannot explain why two diets with similar performance produce different long-term intestinal, immune, metabolic, or stress-resilience outcomes [2,4,10,11,12,13].

The historical development of fish nutrigenomics was strongly shaped by lipid nutrition and the replacement of fish-oil with vegetable oils or alternative lipid sources. Leaver et al. [3] emphasized that fish lipid metabolism is controlled by interactions among dietary fatty acids, nuclear receptors, desaturases, elongases, lipid transport proteins, endocrine factors, and tissue-specific metabolic priorities. Subsequent studies showed that dietary lipid sources can regulate *ppara*, *srebp1*, *fas*, *cpt1*, *fads2*, *elovl5*, apolipoproteins, lipid transporters, and β-oxidation-related genes, particularly in liver, intestine, and muscle [3,14,15,16]. These responses are important because fish oil replacement may maintain short-term growth while altering tissue fatty-acid composition, hepatic lipid deposition, endogenous long-chain polyunsaturated fatty-acid (LC-PUFA) biosynthesis, inflammatory balance, and fillet nutritional value [3,10,14,16].

Alternative-protein sources represent another major driver of fish nutrigenomics. Sustainable aquafeed development increasingly depends on replacing marine fishmeal with soybean meal, soybean-protein concentrate, plant-protein concentrates, insect meals, single-cell proteins, fermented ingredients, microbial biomass, and microalgae [6,9,17,18,19,20]. However, plant-derived proteins may introduce saponins, phytate, non-starch polysaccharides, protease inhibitors, antigenic proteins, and altered amino-acid profiles that affect intestinal morphology, nutrient transport, bile-acid metabolism, mucosal immunity, and intestinal microbiota [7,8,9,17,18,21,22]. Transcriptomic and quantitative polymerase chain reaction (qPCR) endpoints can reveal subclinical intestinal stress, even when growth performance appears acceptable, making gene expression a valuable complement to feed conversion and weight gain data [7,8,9,17].

Nutrigenomics also provides mechanistic insight into nutritional immunology. Fish immune responses are sensitive to nutrient supply, energy allocation, microbial exposure, environmental stress, and intestinal barrier function [4,23,24,25]. Diet-responsive immune markers commonly include *il1beta*, *il6*, *tnfalpha*, *il10*, *tlr*, *myd88*, NF-κB, *lyz*, complement genes, immunoglobulin-related genes, *mucins*, and tight-junction genes, such as *zo-1*, *occludin*, and *claudins* [17,18,24,25,26]. Functional feed additives, including probiotics, prebiotics, synbiotics, phytobiotics, organic acids, tributyrin, nucleotides, and immunostimulants, often target host–microbiota interactions, mucosal immunity, antioxidant defense, and epithelial integrity rather than serving primarily as macronutrient sources [23,24,26].

Microorganisms are not merely passive indicators of diet quality; they are active biological mediators at the diet–host interface. Fish-associated microbiomes include bacteria, archaea, fungi, viruses, and microbial eukaryotes occupying the gastrointestinal tract, skin, gills, feed, water, and tank biofilms, although most nutritional studies remain dominated by bacterial 16S rRNA gene profiling. These communities can hydrolyze otherwise inaccessible substrates, produce short-chain fatty acids (SCFAs) and other metabolites, modify bile acids, supply vitamins or enzymes, exclude pathogens, and calibrate mucosal immune signaling. Their composition and function are shaped by the host genotype, developmental stage, salinity, temperature, rearing system, feeding history, and distinction between transient digesta-associated organisms and mucosa-associated residents [27,28,29].

Amino acids and micronutrients are increasingly interpreted as signaling molecules rather than only as dietary requirements. Methionine can regulate methyl-group metabolism, antioxidant capacity, amino-acid transport, and TOR/S6K-mediated protein synthesis; lysine is linked to muscle-protein accretion; arginine is associated with nitric oxide metabolism, immunity, and intestinal function; tryptophan can affect serotonin, melatonin, stress physiology, and immunity; and taurine is linked to bile-acid conjugation, osmoregulation, lipid metabolism, antioxidant status, and stress tolerance [2,6,30,31,32,33]. Similarly, vitamin E, vitamin C, selenium, zinc, and vitamin D can regulate redox-sensitive pathways, Nrf2/Keap1 signaling, immune markers, tissue protection, mineral metabolism, and stress resilience [4,6,12,34].

The integration of metabolomics, microbiome profiling, proteomics, and epigenomics with transcriptomics further expands the mechanistic resolution of aquafeed studies. Transcriptomics identifies diet-responsive regulatory pathways, metabolomics captures downstream biochemical phenotypes, microbiota profiling reveals host–microbe–diet interactions, and epigenomics can identify persistent nutritional programming effects [10,11,13,35,36,37]. Multi-omic integration is especially important in climate-resilient aquaculture because dietary strategies may support thermal, hypoxic, salinity, handling, and disease resilience through coordinated regulation of heat-shock proteins, antioxidant genes, metabolic enzymes, immune pathways, osmoregulatory transporters, and long-term transcriptional memory [4,10,13,15,33].

Despite rapid progress, fish nutrigenomics remains methodologically and biologically heterogeneous. Studies differ in species, developmental stage, feeding habit, diet formulation, inclusion level, feeding duration, tissue, analytical platform, reference genome quality, statistical threshold, qPCR validation, and integration with phenotypic outcomes [4,10,12,13,38]. Existing reviews have established the value of omics technologies in aquatic nutrition, but an integrated, pathway-centered account is still needed to connect feed ingredients and functional nutrients with intestinal homeostasis, microbial ecology, systemic metabolism, tissue quality, and environmental resilience.

Accordingly, this structured narrative review critically examines representative evidence concerning how dietary interventions regulate gene expression, microbial ecology, and associated metabolic and physiological pathways in cultured finfish. The review does not attempt exhaustive coverage of every dietary ingredient, nutrient, or cultured finfish species; instead, it uses the best-developed and mechanistically informative areas of the current literature to construct a pathway-centered diet–microbiome–host framework. It aims to: (i) organize the literature around major dietary drivers, including alternative proteins, lipid sources, functional additives, amino acids, vitamins and minerals, and stress-modulating diets; (ii) compare tissue-specific molecular responses across intestine, liver, muscle, and immune-relevant tissues; (iii) examine microbial niches, diet-induced community succession, probiotics, fermented ingredients, microbial metabolites, pathobionts, and pathogen-resistance mechanisms; (iv) integrate host transcriptomic responses with growth, feed efficiency, intestinal morphology, microbiota, immunity, antioxidant status, product quality, and stress resilience; and (v) identify methodological priorities for translating host and microbial biomarkers into precision aquafeed design.

## 2. Scope and Organization of the Review

### 2.1. Literature Identification and Evidence Selection

The literature search and selection process followed a structured narrative review approach. Relevant publications were identified through targeted searches of Web of Science Core Collection, Scopus, PubMed/MEDLINE, CAB Abstracts, Aquatic Sciences and Fisheries Abstracts, and ScienceDirect. Searches were supplemented by backward and forward citation tracking from relevant reviews and primary studies and were updated to 7 July 2026.

The search strategy combined terms describing the study population, dietary intervention, and molecular or microbial outcome. The principal search concepts were: (“cultured finfish” OR fish OR aquaculture) AND (aquafeed OR diet* OR nutrition* OR nutrient* OR “feed ingredient*” OR “alternative protein*” OR “fishmeal replacement” OR “fish oil replacement” OR amino acid* OR vitamin* OR mineral* OR probiotic* OR prebiotic* OR synbiotic* OR phytobiotic* OR “fermented feed*” OR tributyrin) AND (nutrigenomic* OR transcriptom* OR “RNA sequencing” OR RNA-seq OR “gene expression” OR qPCR OR microbiome OR microbiota OR “16S rRNA” OR metagenom* OR metatranscriptom* OR metabolom* OR “host–microbiota interaction*”). Search syntax, field tags, truncation, and phrase searching were adapted to the requirements of each database.

Records were screened first by title and abstract and subsequently by full-text relevance. Studies were considered eligible when they: (i) involved cultured finfish; (ii) evaluated a clearly defined dietary ingredient, nutrient, additive, or feeding intervention; (iii) reported host gene-expression, transcriptomic, microbiome, microbial-function, or integrated multi-omic outcomes; and (iv) provided sufficient experimental and analytical information to interpret the relationship between dietary exposure and molecular, microbial, physiological, or production responses. Microorganism-centered studies were retained when microbial community composition or function was evaluated together with host gene expression, intestinal morphology, metabolic phenotype, pathogen challenge, disease resistance, or another biologically relevant outcome.

Studies were excluded when they: (i) focused exclusively on shellfish, crustaceans, other non-finfish species, in vitro systems, or model organisms outside the review scope; (ii) lacked a clearly defined dietary intervention; (iii) addressed environmental toxicology without a dietary component; (iv) reported only descriptive microbial surveys without direct relevance to aquafeed nutrition; (v) lacked interpretable host molecular or microbial outcomes; (vi) provided insufficient experimental or analytical information; or (vii) were available only as conference abstracts, non-peer-reviewed reports, or incomplete publications.

The database searches identified 412 records, and an additional 36 records were identified through backward and forward citation tracking, as well as the reference lists of relevant background reviews, resulting in 448 records. After 121 duplicate records were removed, 327 titles and abstracts were screened. Of these, 258 records were excluded because they did not meet the predefined scope of the review. Sixty-nine full-text reports were sought for retrieval; two reports could not be retrieved, and 67 full-text reports were assessed for eligibility.

Twenty-four full-text publications were excluded because they focused on shellfish, crustaceans, non-finfish species, in vitro systems, or model organisms outside the review scope (*n* = 5); lacked a clearly defined dietary intervention (*n* = 4); addressed environmental toxicology without a dietary component (*n* = 3); reported only descriptive microbiome surveys without direct relevance to aquafeed nutrition (*n* = 3); lacked interpretable host molecular or microbial outcomes (*n* = 3); provided insufficient experimental or analytical information (*n* = 3); were available only as conference abstracts, non-peer-reviewed reports, or incomplete publications (*n* = 2); or were otherwise not directly relevant to cultured finfish precision aquafeed development (*n* = 1).

**Figure 1:**
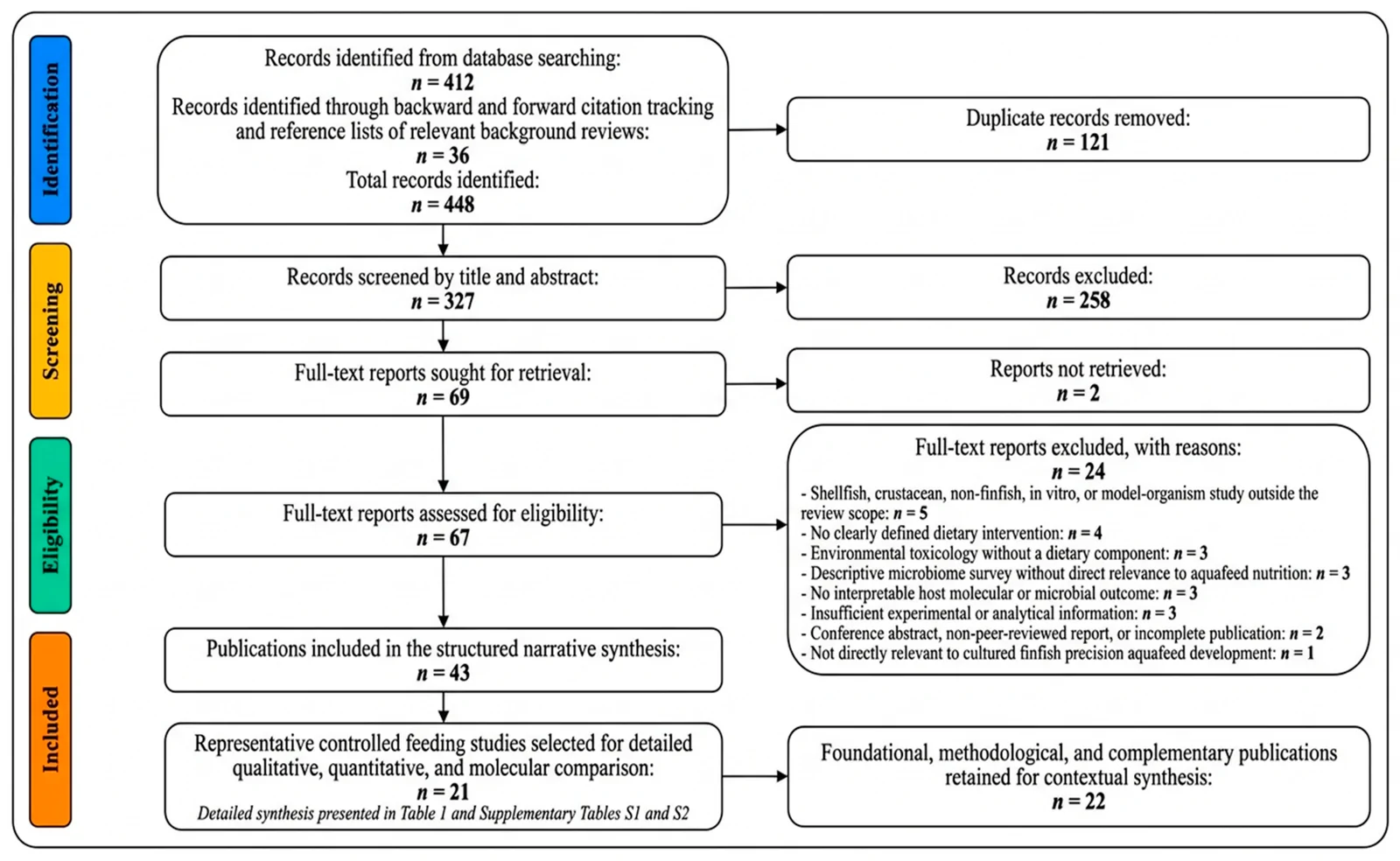
Literature-identification and evidence-selection workflow for the structured narrative review. Publications were identified through targeted searches of Web of Science Core Collection, Scopus, PubMed/MEDLINE, CAB Abstracts, Aquatic Sciences and Fisheries Abstracts, and ScienceDirect, supplemented by backward and forward citation tracking. After duplicate removal, records were screened by title and abstract and subsequently assessed according to the full-text eligibility criteria. Forty-three publications were retained in the structured narrative synthesis. Of these, 21 representative controlled feeding studies were selected for detailed study-design, quantitative-outcome, and molecular-method comparison in Table 1, Tables S1 and S2, whereas 22 foundational, methodological, and complementary publications were retained to provide biological and analytical context. The arrows indicate only the sequential identification, screening, eligibility, and inclusion stages and do not represent biological or causal relationships.

A total of 43 publications were retained in the structured narrative synthesis. Of these, 21 representative controlled feeding studies were selected for detailed study-design, quantitative-outcome, and molecular-method comparison in Table 1, Tables S1 and S2 based on their mechanistic relevance, methodological diversity, and coverage of the major dietary and microorganism-centered themes. The remaining 22 publications comprised foundational reviews, methodological guidelines, and complementary molecular, microbial, and multi-omic studies retained to provide necessary biological and analytical context. The literature-identification and evidence-selection process is summarized in Figure 1. No new or unpublished experimental data are presented.

To facilitate transparent quantitative comparison without implying a formal meta-analysis, study-level information was extracted from the 21 representative feeding-study reports summarized in Table 1. Extracted experimental-design variables included species, initial body sizes, dietary inclusion or replacement levels, feeding durations, number of independent tanks or cages, fish per experimental unit, and the number of biological samples or molecular libraries. Extracted analytical variables included sampled tissue, molecular platform, pooling structure, read length, sequencing depth or sequence yield, mapping and quality metrics, differential-expression criteria, fold change or log2 fold change, statistical threshold, and data-accession information when reported. Extracted outcomes included growth, feed utilization, survival, biochemical and enzyme responses, histological observations, microbial outcomes, challenge responses, and molecular endpoints. Where control and treatment means were available in directly comparable units, the relative difference was calculated as [(treatment − control)/control] ×100 and interpreted as a descriptive within-study effect magnitude rather than a standardized effect size. Standardized mean differences were not calculated consistently because variance estimates, directly comparable endpoint definitions, and independent experimental-unit sample sizes were not uniformly available. Numerical values were transcribed from the original articles; information that was presented only graphically, not reported, or not directly comparable was identified accordingly and not estimated. Study-design characteristics, experimental units, analytical methods, and principal limitations are presented in Section 3.1. Detailed dietary comparisons and quantitative production, physiological, histological, molecular, and microbiome outcomes are provided in Supplementary Table S1. Tissue-specific molecular endpoints, analytical platforms, library construction or pooling, sequencing scale and quality, statistical criteria, and data-accession status are summarized in Supplementary Table S2. The extracted data were not statistically pooled because of substantial biological and methodological heterogeneity.

### 2.2. Thematic and Pathway-Centered Framework

Evidence was organized by dietary domain and then interpreted through cross-cutting biological pathways. The major dietary domains were alternative-protein sources, lipid sources, and long-chain polyunsaturated fatty-acid supply; functional feed additives and host–microbiota interactions; amino acids, vitamins, and minerals; and stress-related nutritional programming. Molecular and microbial responses were grouped into lipid metabolism, amino-acid transport and growth signaling, intestinal inflammation and barrier integrity, nutrient transport, antioxidant and stress responses, bile-acid metabolism, microbial succession, probiotic and pathobiont activity, microbial metabolite signaling, and epigenetic or developmental programming.

Gene-expression findings were interpreted together with protein abundance or activity, enzyme activity, metabolite concentrations, growth, feed conversion, feed efficiency, tissue composition, intestinal histology, microbiota, immune and antioxidant responses, disease or parasite outcomes, and environmental stress tolerance. This integrative approach avoids treating the direction of a single transcript as inherently beneficial or adverse, and instead emphasizes the tissue context, dose, species, life stage, and concordance among transcriptional, biochemical, histological, and phenotypic outcomes.

Because most reviewed studies measured molecular, microbial, and phenotypic endpoints concurrently, co-occurring responses were interpreted as associations unless temporal precedence, experimental perturbation, mediation analysis, microbial transplantation, defined-community testing, or another direct causal design was used.

To standardize the strength of interpretation, the reviewed findings were assigned to one of four descriptive evidence levels. Level 1—Replicated controlled evidence that indicates support from several independent controlled feeding studies, together with concordant molecular and downstream functional or phenotypic outcomes. Level 2—Integrated but limited-replication evidence that indicates support principally from one integrated controlled study, or from a small evidence base with limited independence, but with more than one molecular, biochemical, microbial, histological, or phenotypic evidence layer. Level 3—Molecular or marker-based evidence that indicates support mainly from gene-expression, transcriptomic, 16S rRNA, taxonomic-association, or predicted-function data without direct functional or causal validation. Level 4—A hypothetical or proposed mechanism that identifies biologically plausible relationships derived from cross-study synthesis or related biological systems that have not been demonstrated directly in cultured finfish. These categories are descriptive synthesis tools and do not constitute formal risk-of-bias or study-quality grades.

## 3. Dietary Interventions and Nutrigenomic Responses

### 3.1. Representative Evidence Landscape

Representative controlled feeding studies span freshwater, euryhaline, and marine species and include Japanese seabass, yellow perch, groupers, grass carp, rainbow trout, gilthead sea bream, golden pompano, common carp, cobia, largemouth bass, Senegalese sole, *Sillago sihama*, olive flounder, tilapia, and gray mullet. The intestine is the dominant tissue in studies of alternative proteins, probiotics, and functional additives, whereas liver and muscle are frequently used to resolve lipid, amino-acid, endocrine, and myogenic responses.

The evidence now includes targeted qPCR panels, microarrays, RNA-seq, full-length transcriptomics, small RNA sequencing, 16S rRNA community profiling, pathogen challenge, and integrated host-transcriptome–microbiome or multi-omic designs. Representative controlled feeding studies covering the major nutrigenomic and microorganism-centered themes are summarized in Table 1, with an emphasis on study designs, experimental units, analytical methods, and principal methodological limitations.

Detailed dietary comparisons and quantitative production, physiological, histological, molecular, and microbiome outcomes are provided in Supplementary Table S1. Supplementary Table S2 presents the corresponding tissue-specific molecular endpoints, analytical platforms, library construction or pooling, sequencing scale and quality, statistical criteria, and public data availability information.

**Table 1:** Study-design characteristics, experimental units, analytical methods, and principal limitations of representative controlled feeding studies in cultured finfish.

Species	Dietary Intervention	Feeding Period and Stocked Design	Molecular/Functional Sampling	Experimental Unit	Principal Analytical Methods	Principal Methodological Limitation	References
Japanese seabass (*Lateolabrax japonicus*)	Fishmeal control; 50% or 75% fish-meal replacement with soybean meal.	8 weeks; 3 tanks/treatment; 20 fish/tank; IBW 6.67 ± 0.03 g.	Separate 3 fish/tank sets for histology, digestive enzymes, RNA, and serum analyses; qPCR technical triplicates.	Tank.	SYBR RT-qPCR of inflammatory and nutrient-transporter genes; digestive enzymes; intestinal histology.	Transcript fold changes were graphical, and statistical handling of fish subsamples nested within tanks was unclear.	[17]
Yellow perch (*Perca flavescens*)	Fishmeal control versus 75% replacement of fish-meal protein with soybean meal.	61 days; 6 tanks/treatment; 50 fish/tank; IBW 0.13 ± 0.03 g.	One fish/tank; 6 RNA-seq libraries/diet (12 total); same samples used for qPCR validation.	Tank.	Illumina HiSeq 3000/4000 RNA-seq (150 bp paired-end), de novo Trinity assembly, edgeR, and qPCR.	Public accession was not stated in the reviewed main article; the first-formulated-feed juvenile context limits generalization.	[7]
Hybrid grouper (*E. fuscoguttatus* ♀ *× E. lanceolatus* ♂)	0%, 10%, 30%, or 50% fishmeal-protein replacement with soybean meal.	10 weeks; 3 tanks/treatment; 30 fish/tank; IBW 17.01 ± 0.04 g.	Histology: 2 fish/tank; three mRNA and three small-RNA replicates/diet, each pooling distal intestine from 3 fish/tank; same samples used for qPCR.	Tank; pooled fish formed one biological replicate.	BGISEQ-500 mRNA and small-RNA sequencing, DESeq, integrated miRNA-mRNA analysis, and histology.	Omic analyses compared only FM and the 50% replacement group; pooling prevented fish-level independence.	[18]
Pearl gentian grouper (*E. fuscoguttatus* ♀ *× E. lanceolatus* ♂)	Fishmeal control; 20% or 40% fish-meal-protein replacement with fermented soybean meal.	10 weeks; 4 barrels/treatment; 60 fish/barrel; IBW 12.55 g.	Distal intestine from 4 fish/barrel; 4 tank-level replicates; exact sequencing-library pooling was incompletely described.	Barrel/tank.	PacBio Sequel full-length transcriptomics with Illumina correction, RT-qPCR, intestinal histology, and biochemical assays.	Sequencing depth and pooling were not fully reported; closely related to studies 5–6 and used previously generated SBM40 data.	[20]
Pearl gentian grouper (*E. fuscoguttatus* ♀ *× E. lanceolatus* ♂)	Fishmeal control; 20% or 40% fish-meal-protein replacement with soybean meal.	10 weeks; 4 barrels/treatment; 60 fish/barrel; IBW 12.55 g.	Transcriptomics: 4 replicates/treatment; 16S: 3 samples/group; metabolomics: 12 pooled intestinal-content samples/treatment, 3 fish/pool.	Tank for host outcomes; omics sampling was tank-derived, with pooling reported for metabolomics.	Full-length transcriptomics, 16S rRNA sequencing, LC-MS metabolomics, RT-qPCR, and Western blot.	Host-transcriptome depth was not reported; transcriptome and 16S replicate construction was incompletely described.	[22]
Pearl gentian grouper (*E. fuscoguttatus* ♀ *× E. lanceolatus* ♂)	Fishmeal control; 20% or 40% fish-meal-protein replacement with soybean-protein concentrate.	10 weeks; 4 barrels/treatment; 60 fish/barrel; IBW 12.55 g.	Six fish/barrel for biochemical and gene-expression analyses; eight additional fish/barrel for 16S and transcriptome sequencing (four each); three fish/barrel for histology; exact library pooling unclear.	Barrel/tank.	PacBio/Illumina full-length transcriptomics, 16S rRNA sequencing, RT-qPCR, Western blot, and histology.	Exact sequencing-library pooling and numerical quality/mapping metrics were not reported; the Illumina accession appears incomplete, and downregulated-DEG counts were internally inconsistent.	[21]
Grass carp (*Ctenopharyngodon idellus*)	Complete soybean-meal replacement with *C. autoethanogenum* protein, Tenebrio meal, cottonseed-protein concentrate, or *Chlorella* powder; two fish sizes.	56 days; 3 cages/treatment/size; 25 fish/cage; IBW 239.72 or 638.32 g.	Six fish per protein source and size class; qPCR and amino-acid analyses reported *n* = 3, and texture analysis *n* = 6.	Cage.	ABI 7500 RT-qPCR of *TOR* and myogenic genes; muscle composition, texture, and quality analyses.	Size × diet interactions complicate generalization; many numerical differences were not significant, and molecular analysis was targeted to muscle.	[19]
Rainbow trout (*Oncorhynchus mykiss*)	Marine diet, commercial-like partially plant-based diet, or fully plant-based diet from first feeding.	13 months; first 7 months: 4 tanks/diet, 310 fry/tank; next 6 months: 3 tanks/diet, 150 fish/tank; IBW 0.135 g.	Microarray: 8 fish/diet/tissue (48 arrays); qPCR: 6 fish/tissue/condition at each stage.	Tank for performance; individual fish sampled across tanks for molecular assays.	Custom Agilent 8 × 60K microarray and RT-qPCR; growth, composition, and fatty-acid analyses.	Sequential life stages and temperature regimes mean the experiment was not one uniform exposure period.	[14]
Gilthead sea bream (*Sparus aurata*)	Broodstock selected for high/low-blood *fads2* expression and fed fish oil or high-vegetable-oil diets; offspring challenged with low-FM/FO diet.	Broodstock conditioning plus 2-month offspring challenge; 3 offspring tanks/group.	Seventy broodstock screened; offspring ddPCR *n* inconsistently reported (9 vs. 3/group); methylation, *n* = 3–5/group; liver fatty acids, 5 pooled livers/tank.	Offspring tank for feeding; individual or pooled samples for molecular endpoints.	RT-qPCR, droplet digital PCR, and *fads2*-promoter pyrosequencing.	Complex parental-selection/programming design, small methylation subgroups, and several graphically reported outcomes limit direct comparison.	[15]
Golden pompano (*Trachinotus ovatus*)	Five diets containing 0.64%, 1.00%, 1.24%, 1.73%, or 2.10% n-3 HUFA.	56 days; 3 cages/treatment; 25 fish/cage; IBW 10 g.	Muscle from 6 fish/cage; qPCR used 2 fish/cage × 3 cages; RNA-seq library number and pooling were unclear.	Cage.	NovaSeq 6000 RNA-seq, de novo Trinity assembly, RT-qPCR, and detailed TAG/phospholipid fatty-acid analysis.	Growth and feed-utilization outcomes were not reported; library replication, mapping metric, and public accession were insufficiently clear.	[16]
Common carp (*Cyprinus carpio*)	Fishmeal control and all-plant diets supplemented with 0.5, 1.0, 2.0, or 4.0 g/kg tributyrin.	8 weeks; 3 tanks/treatment; 30 fish/tank; IBW approximately 8.1 g.	Six fish/tank for serum and intestinal functional/qPCR analyses; 16S used contents pooled from 3 fish/tank, yielding 3 pooled samples/treatment.	Tank.	Targeted RT-qPCR, Illumina 16S V3-V4 sequencing, digestive enzymes, and intestinal histology.	No unsupplemented all-plant group; sequence depth was not reported, and microbial functions were inferred from 16S data.	[26]
Gilthead sea bream (*S. aurata*)	Conventional control versus fishmeal-free circular diet containing alternative proteins, oils, and a health additive.	34-day pre-challenge phase: 160 fish/diet in one large tank; parasite phase: 3 tanks/diet; IBW 21.3 g.	Eighteen fish total (9/diet) used for qPCR arrays and adherent-microbiota analysis.	One tank/diet pre-challenge; individual fish served as replicates during challenge after no tank effect was detected.	Customized qPCR arrays, MiSeq 16S V3-V4 sequencing, growth assessment, and intestinal parasite challenge.	Pre-challenge growth, gene-expression, and microbiota comparisons lacked tank-level replication; FCR was a whole-tank value.	[24]
Gilthead sea bream (*S. aurata*)	Reference versus growth-selected fish fed control or FUTURE low-marine-resource diets across a production cycle.	12 months; 20 tanks total; 5 tanks/group; 50 fish/tank (250 fish/group); sampling at approximately 10, 200, and 350 g.	16S: 94 individual fish across time points; RNA-seq: 36 individual libraries at the intermediate stage.	Tank for the 2 × 2 genotype × diet feeding design; individual fish were sampled for molecular analyses.	MiSeq 16S V3-V4 sequencing, HiSeq 2500 RNA-seq (2 × 150 bp), DESeq2, and host-transcript/OTU association analysis.	Longitudinal genotype × diet design; detailed growth outcomes were in a companion study, and no single treatment-effect estimate applies.	[25]
Cobia (*Rachycentron canadum*)	Seven low-fish-meal diets containing 0.72–1.86% methionine.	8 weeks; 3 sea cages/treatment; 40 fish/cage; IBW 9.79 ± 0.04 g.	Tissues from 3 fish/cage pooled; 3 cage-level biological replicates/treatment.	Cage.	SYBR RT-qPCR and Western blot of phosphorylated TOR, S6K, and 4E-BP1; growth and requirement regression.	Pooled samples and a targeted pathway panel limit fish-level inference and broader mechanistic interpretation.	[30]
Largemouth bass (*Micropterus salmoides*)	Adequate methionine (1.21%) versus methionine-deficient diet (0.93%).	8 weeks; 3 tanks/treatment; 20 fish/tank; IBW 4.10 ± 0.01 g.	Four fish/tank sampled and pooled at tank level; 3 biological replicates/treatment.	Tank.	SYBR RT-qPCR and Western blot/phosphoprotein analysis of nutrient sensing and amino-acid transport.	Two-diet design limits dose–response inference; pooling limits fish-level inference; transcript values were shown graphically.	[31]
Senegalese sole (*Solea senegalensis*)	Fishmeal diet, plant-protein diet, and plant diets supplemented with 0.5% or 1.5% taurine.	6 weeks; 3 tanks/treatment; 35 fish/tank; IBW approximately 14 g.	Three liver samples/tank (9 fish/treatment); reverse transcription and qPCR performed in duplicate.	Tank.	StepOnePlus RT-qPCR of bile-acid/lipid genes; tissue taurine, bile acids, lipid deposition, and plasma biochemistry.	Several expression outcomes were graphical; targeted liver genes and the 6-week duration limit conclusions about long-term pathway activity.	[32]
Silver sillago (*Sillago sihama*)	Six diets containing 4, 14, 22, 47, 95, or 198 mg/kg vitamin E.	8 weeks; 4 tanks/treatment; 30 fish/tank; IBW 2.14 ± 0.02 g.	Expression figures reported 16 fish/treatment: 4 fish from each of 4 replicate tanks.	Tank.	ABI 7500 SYBR RT-qPCR of *nrf2*/*keap1* and antioxidant genes; growth, immune, antioxidant-enzyme, and MDA assays.	Gene-expression values were graphical, and protein/pathway activity was not measured directly; requirement estimates depended on endpoint.	[34]
Olive flounder (*Paralichthys olivaceus*)	Seven diets containing 0.13–2.87% taurine, followed by acute 29 °C and lethal 31 °C thermal tests.	8 weeks; 3 tanks/treatment; 20 fish/tank; IBW 12.97 ± 0.10 g.	Three fish/tank were exposed and sampled; molecular results reported from 3 tank-level replicates per diet × temperature condition; blood pooled by tank.	Tank.	StepOne RT-qPCR of *hsp60*, *hsp70*, *hsp90*, and *wap65*; plasma/antioxidant assays and thermal-survival challenge.	Stress effects were larger than dietary effects; blood pooling reduced fish-level resolution and taurine did not improve lethal-stress survival.	[33]
Tilapia (*Oreochromis* spp.)	Control diet versus 0.5% or 1.0% *Bidens pilosa*.	8 weeks; 3 net cages/treatment; 15 fish/cage; IBW 7.5 ± 1.0 g.	Blood: 3 fish/cage (9/treatment); transcriptome: 15 fish (5/group), but only 6 SRA runs deposited; microbiome *n* = 3/group.	Net cage for growth and blood outcomes; transcriptome-library unit unclear.	Short-read RNA-seq, qRT-qPCR, Oxford Nanopore full-length 16S sequencing, growth, and serum biochemistry.	The relationship between 15 fish and 6 RNA-seq runs was unclear; microbiome sequencing yield/read counts were not reported.	[39]
Hybrid grouper (*E. fuscoguttatus* ♀ *× E. lanceolatus* ♂)	Control; *B. cereus*; *E. acetylicum*; 1:1 mixed strains; *L. acidophilus* positive reference.	60 days; 4 tanks/treatment; 30 fish/tank; IBW 53.30 ± 0.50 g.	Three fish/tank collected for tissues; gene data reported as *n* = 4 replicates, but handling of the three fish/tank was not specified; microbiota used 4 fish/group.	Tank for growth; qPCR and microbiota experimental units were unclear.	LightCycler RT-qPCR, MiSeq 16S V4–V5 sequencing, digestive enzymes, immunity/antioxidant assays, and taxon–host correlations.	Sequence depth and accession were not reported; exact growth/qPCR means were graphical, and probiotic safety was not comprehensively assessed.	[40]
Gray mullet (*Mugil cephalus*)	Control versus *L. rhamnosus* FS3051, *L. reuteri* FS3052, or *B. subtilis* natto NTU-18 at 10^9^ CFU/g diet.	28 days; growth/gene/microbiome phase: 1 tank with 6 fish/group; challenge phase: 3 tanks × 10 fish/group; IBW 1.07 ± 0.07 g.	qPCR: 6 fish/group from the single tank; microbiome: 3 fish/group for control and *L. rhamnosus* only; challenge: 3 tanks/group.	Individual fish were observations within one tank for growth/qPCR; challenge survival pooled 30 fish across three tanks/group.	MyiQ RT-qPCR of liver/spleen immune genes, HiSeq 16S V3–V4 sequencing, and *Nocardia seriolae* immersion challenge.	Growth, qPCR, and microbiome phases were pseudoreplicated because each diet had one tank; microbiome compared only two groups, and no public accession was stated.	[41]

Abbreviations: 4E-BP1, eukaryotic translation initiation factor 4E-binding protein 1; 16S rRNA, 16S ribosomal RNA; bp, base pair; CFU, colony-forming unit; ddPCR, droplet digital polymerase chain reaction; DEG, differentially expressed gene; *fads2*, fatty-acid desaturase 2; FCR, feed conversion ratio; FM, fishmeal; FO, fish oil; *hsp60*, *hsp70*, and *hsp90*, heat-shock proteins 60, 70, and 90, respectively; IBW, initial body weight; LC-MS, liquid chromatography–mass spectrometry; MDA, malondialdehyde; miRNA, microRNA; mRNA, messenger RNA; n-3 HUFA, omega-3 highly unsaturated fatty acid; Nrf2, nuclear factor erythroid 2-related factor 2; Keap1, Kelch-like ECH-associated protein 1; NR, not reported; OTU, operational taxonomic unit; qPCR, quantitative polymerase chain reaction; qRT-qPCR/RT-qPCR, quantitative reverse-transcription polymerase chain reaction; RNA-seq, RNA sequencing; S6K, ribosomal protein S6 kinase; SRA, Sequence Read Archive; TAG, triacylglycerol; TOR, target of rapamycin; V3–V4 and V4–V5, hypervariable regions of the 16S rRNA gene; *wap65*, warm-temperature acclimation-related 65 kDa protein. Footnote: Sample numbers refer to the molecular or functional component and may differ from the total number of stocked fish. Studies 4–6 are closely related reports and may contain overlapping or reused control data; they should not be treated as fully independent effect estimates. “Experimental unit” identifies the unit that received the dietary treatment or the unit appropriate for independent inference; subsampled fish within one tank are not independent dietary replicates.

The study-level comparison in Supplementary Table S1 shows that the magnitude and direction of dietary effects varied widely among species and experimental contexts. High soybean-derived protein replacement generally reduced growth or feed utilization and intensified intestinal injury in carnivorous finfish, whereas moderate replacement or processed ingredients produced smaller or context-dependent effects. Nutrient and functional-additive studies more often showed dose-dependent optima, but transcriptional responses were not uniformly accompanied by growth, biochemical, histological, or challenge improvements. Several investigations reported mechanistic endpoints without directly comparable production data, and some numerical outcomes were available only in figures. Accordingly, the values in Supplementary Table S1 should be interpreted as within-study descriptive comparisons rather than as pooled estimates of the efficacy of a dietary strategy.

The molecular analytical scale also varied substantially among the representative studies. The yellow perch RNA sequencing study generated approximately 68 million passing-filter reads per sample [7], whereas the hybrid grouper messenger RNA libraries contained 72.38–76.50 million clean reads per library, with total mapping rates of 75.76–79.02% [18]. The fishmeal-free gilthead sea bream microbiome study generated 4,068,405 high-quality merged reads across 18 samples, averaging 226,022 reads per sample [24]. In the production-cycle gilthead sea bream study, approximately 10 million high-quality 16S ribosomal RNA reads were obtained from 94 fish, while 36 host RNA sequencing libraries averaged approximately 28 million paired-end reads per sample and mapped at rates of up to 93% [25]. Reported molecular-response magnitudes ranged from at least threefold differences in 33 genes in yellow perch [7] to 6390 differentially expressed genes and 92 differentially expressed microRNAs in hybrid grouper [18]. Other studies reported exact quantitative polymerase chain reaction fold means, targeted protein phosphorylation, or only graphical expression outcomes. Quantitative molecular-response outcomes are reported in Supplementary Table S1, whereas Supplementary Table S2 presents library construction or pooling; sequencing scale, quality, and mapping metrics, molecular or statistical criteria; and public data-accession status. Unavailable or graphically reported information was not estimated.

### 3.2. Alternative-Protein Sources and Intestinal Tolerance

Alternative-protein sources represent a major application of finfish nutrigenomics. However, within the evidence base retained for this review, most direct molecular and multi-omic evidence concerns soybean meal, fermented soybean meal, and soybean-protein concentrate. Controlled nutrigenomic studies of insect meals, microbial or single-cell proteins, microalgae, and other emerging protein sources remain comparatively limited. The intestine is usually the most responsive tissue because it is directly exposed to antinutritional factors, altered amino-acid profiles, microbial metabolites, bile acids, and mucosal immune stimuli [7,8,9,17,18,22].

In Japanese seabass, replacing 50% or 75% of fishmeal with soybean meal altered intestinal histology, digestive enzymes, inflammatory and transporter genes, growth, and feed utilization [17]. Considered together, these responses indicate reduced intestinal tolerance at high inclusion and may reflect soybean antinutritional factors acting through NF-κB-related inflammatory and barrier pathways [8,9,17,18].

Accordingly, inflammatory- and transporter-gene responses were interpreted together with digestive enzyme activity, intestinal histology, growth, and feed utilization rather than as independent evidence of impaired intestinal function [17].

Saponins, phytate, non-starch polysaccharides, antigenic proteins, and altered amino-acid profiles have been proposed to influence pattern-recognition receptor and NF-κB-related signaling, cytokine transcription, and epithelial-barrier remodeling. In the reviewed studies, the observed transcriptional and histological responses were consistent with potential involvement of these processes rather than direct demonstration of pathway activation [8,9,17,18].

Yellow perch showed a more selective response to soybean meal as the first formulated feed. Kemski et al. [7] reported increased expression of genes associated with intestinal cholesterol biosynthesis, suggesting a focused transcriptional adjustment involving sterol metabolism rather than demonstrated activation of the complete pathway.

This finding is important because it shows that alternative-protein sources can affect lipid and sterol metabolism in the intestine, even when the intervention is primarily framed as a protein replacement. It also reinforces the need to interpret intestinal transcriptomes not only through inflammation but also through membrane lipid synthesis, bile-acid-related processes, epithelial renewal, and nutrient transport [7,32].

Hybrid and pearl gentian grouper studies further showed that poorly tolerated soy-protein inclusion was associated with enteritis-related immune regulation and disrupted intestinal homeostasis [8,18,22]. Fermentation moderated some responses, while excessive soybean-protein concentrates still induced enteritis, supporting dose-, processing-, and species-dependent tolerance [8,9,18,22].

Evidence for non-soy alternative proteins remains more limited. Fan et al. [19] compared complete replacement of soybean meal with CAP, *Tenebrio molitor* meal, cottonseed-protein concentrate, or *Chlorella* powder in two size classes of grass carp. Growth and feed-utilization responses varied with ingredient and fish size, and the numerical differences from the soybean-meal treatment were not consistently significant. *Chlorella* powder was associated with favorable muscle composition and flesh-quality traits and altered TOR/S6K- and myogenic-gene expression in smaller fish, whereas the responses were less evident in larger fish.

This study demonstrates that insect, microbial, plant-concentrate, and microalgal proteins may produce ingredient- and life-stage-specific molecular and phenotypic responses. Nevertheless, the available evidence remains insufficient for the same level of mechanistic synthesis currently possible for soybean-derived proteins. Future studies should evaluate these emerging proteins using coordinated intestinal, hepatic, muscular, microbial, and production-level endpoints [19,31].

### 3.3. Lipid Sources, LC-PUFA Biosynthesis, and Tissue Quality

Lipid nutrigenomics addresses fish oil replacement, dietary n-3 highly unsaturated fatty-acid (n-3 HUFA) supply, endogenous long-chain polyunsaturated fatty-acid biosynthesis, tissue lipid deposition, and nutritional programming. Core molecular markers include *fads2*, *elovl5*, *ppara*, *cpt1*, *srebp1*, *fas*, lipid transporters, and genes involved in triacylglycerol and phospholipid metabolism [3,14,15,16].

Lazzarotto et al. [14] provided a long-term rainbow trout model in which fishmeal and fish oil were replaced with plant-based ingredients from first feeding. This study is particularly valuable because it linked growth and whole-body fatty acids with intestinal and hepatic gene expression. Long-term dietary exposure is important in lipid nutrigenomics because short-term transcriptional compensation may not accurately predict cumulative tissue fatty-acid deposition, hepatic lipid accumulation, or flesh nutritional quality [10,14].

Türkmen et al. [15] extended lipid nutrigenomics into nutritional programming by examining dietary lipid composition and *fads2* expression in gilthead sea bream. This study is mechanistically important because *fads2* encodes a key desaturase involved in LC-PUFA biosynthesis, and its regulation can be influenced by both diet and genetic/epigenetic factors. In the context of precision aquafeeds, *fads2* is not only a marker of immediate lipid metabolism but also a potential selection and programming target for improving utilization of vegetable oils and maintaining eicosapentaenoic acid (EPA) and docosahexaenoic acid (DHA) deposition when marine lipid resources are reduced [3,13,15].

Gao et al. [16] provided recent evidence in golden pompano that graded dietary n-3 HUFAs affect muscle fatty-acid composition, triacylglycerol positional distribution, phospholipid composition, and lipid-metabolism gene expression. This is important because nutritional value depends not only on the total content of EPA and DHA but also on lipid class and stereospecific positioning within triacylglycerols and phospholipids. Therefore, lipid nutrigenomics should increasingly integrate transcriptomics with detailed lipidomics, tissue fatty-acid composition, growth, and fillet-quality outcomes [10,11,16].

Accordingly, lipid-related transcriptional responses were interpreted together with tissue fatty-acid composition, lipid-class distribution, EPA and DHA deposition, growth, and fillet quality. Transcriptional compensation without restoration of these downstream outcomes was not considered evidence of functional recovery [10,11,14,15,16].

### 3.4. Functional Feed Additives and Host–Microbiota Interactions

Functional feed additives should not be treated as a single mechanistic class. For clarity, they are organized here according to their predominant mode of action: live microbial interventions, microbiota-directed substrates, metabolite- or postbiotic-based strategies, plant-derived bioactives, and fermented ingredients or microbial biomass. Although these categories may share downstream effects, they differ in their primary modes of action and therefore require category-specific analytical endpoints and validation approaches [23,24,25,26,39,40,41].

Live microbial interventions, including probiotics and synbiotics, depend on the delivery of viable, strain-defined microorganisms. Their potential actions include digestive-enzyme production, competition for nutrients and adhesion sites, antimicrobial-product formation, immune modulation, and restructuring of resident microbial communities. In hybrid grouper, single- and mixed-strain preparations produced different growth, digestive-enzyme, antioxidant, immune-gene, and microbiota responses [40]. In gray mullet, probiotic effects were also strain specific, and *Lacticaseibacillus rhamnosus* improved survival following *N. seriolae* challenge [41]. These interventions therefore require strain-level identification, viable-dose verification, feed-stability testing, biosafety assessment, and challenge validation.

Microbiota-directed substrates, including prebiotics, act primarily by providing selectively utilized substrates rather than viable microorganisms. Their evaluation should focus on substrate utilization, absolute microbial changes, fermentation products, intestinal-barrier responses, and host phenotypes. Direct prebiotic evidence was less extensively represented than probiotic and tributyrin studies in the literature retained for this review; therefore, broad efficacy claims were avoided.

Metabolite- and postbiotic-based strategies, including tributyrin, organic acids, and microbial-derived products, act through delivered metabolites or microbial components rather than through colonization by viable organisms. Tributyrin supplementation in common carp was associated with intestinal morphology, barrier-related gene expression, and microbial-community changes [26]. These strategies should be evaluated using direct metabolite measurements, intestinal histology, barrier and immune markers, and microbial outcomes.

Plant-derived bioactives or phytobiotics may influence endocrine, metabolic, antioxidant, immune, and microbial responses through plant secondary compounds. Dietary *Bidens pilosa* in tilapia was associated with growth, host transcriptomic responses, and intestinal microbial profiles [39]. However, microbial functions predicted from marker-gene data remained inferential and required direct functional validation.

Fermented ingredients and microbial biomass are distinct from live probiotics. Fermentation may reduce antinutritional factors and release peptides, organic acids, enzymes, and microbial cell-wall components, whereas microbial biomass may act as a protein source, nucleotide source, enzyme carrier, or bioactive material. Their evaluation should therefore report the production organism, residual viability, metabolite profile, cell-wall composition, digestibility, and batch consistency.

Complex functional-feed formulations may combine several of these mechanisms. Piazzon et al. [24] linked a fishmeal-free functional formulation with growth, tissue-specific gene expression, microbiota, and parasite susceptibility, whereas Naya-Catala et al. [25] demonstrated that the host genotype and production stage modified diet–microbiota–transcriptome interactions. Such formulations should therefore be evaluated using integrated molecular, microbial, physiological, and challenge-related outcomes rather than being attributed to a single additive mechanism.

### 3.5. Amino Acids as Nutrients and Metabolic Signals

Amino-acid nutrigenomics is particularly relevant to low-fishmeal and plant-based aquafeeds, in which methionine, lysine, taurine, and other nutrients may become limiting or biologically imbalanced. Beyond serving as substrates for protein synthesis, amino acids regulate nutrient transporters, TOR/S6K and IGF signaling, methyl-group metabolism, antioxidant capacity, bile-acid conjugation, lipid utilization, osmoregulation, and stress physiology [6,30,31,32].

He et al. [30] reported that DL-methionine supplementation in a low-fishmeal diet altered the transcript abundance of *TOR*, *S6K*, *ASCT2*, and *IGF-I* in the dorsal muscle of juvenile cobia. These transcriptional responses were accompanied by phosphoprotein measurements of TOR, S6K, and 4E-BP1, with TOR Ser2448 phosphorylation highest at 1.24% dietary methionine. Together with growth and metabolic outcomes, these findings support coordinated responses involving amino-acid transport, nutrient sensing, endocrine growth signaling, and muscle-protein accretion, although the pooled samples and targeted pathway panel limit broader mechanistic inference [30].

In this context, methionine functions not only as a limiting amino acid for protein deposition but also as a metabolic signal. Its availability can influence TOR, S6K, 4E-BP1, IGF-I, amino-acid transporters, and myogenic endpoints, thereby connecting dietary formulation with anabolic signaling and muscle growth [2,30,31].

Wang et al. [31] extended this concept in largemouth bass by showing that dietary methionine improved growth performance and metabolism through the modulation of nutrient-related pathways. Compared with methionine deficiency, adequate methionine supply improved free amino-acid status, amino-acid transporter efficiency, feed efficiency, and nutrient-sensing signaling.

These findings support a precision-formulation approach in which crystalline amino acids are evaluated not only through growth performance and requirement estimates but also through transporter expression, TOR signaling, protein-use efficiency, and tissue-specific metabolic outcomes. Such measurements can help determine whether supplementation produces coordinated molecular and production-level benefits [6,12,31].

Aragão et al. [32] provided a complementary taurine model in Senegalese sole fed plant-based diets. Plant-protein replacement reduced taurine and bile-acid concentrations and downregulated *cyp7a1* and *abcb11*. Taurine supplementation increased tissue taurine and partially mitigated bile-acid- and lipid-metabolism disturbances; 0.5% taurine increased *cyp7a1* and *abcb11* relative to the non-supplemented plant-protein diet, although full restoration to the fishmeal expression level was not demonstrated. These findings link taurine availability with bile-acid metabolism, lipid utilization, and hepatic health [32,33].

### 3.6. Vitamin and Mineral Nutrigenomics

Compared with macronutrient and alternative-ingredient research, vitamin and mineral nutrigenomics in cultured finfish remains less developed. Within the representative feeding studies included in this review, a dedicated micronutrient dose–response design was represented principally by the vitamin E study in *Sillago sihama* [34]. Evidence concerning vitamin C, selenium, zinc, and vitamin D was derived mainly from broader reviews and the complementary literature [4,6,12] and should not be regarded as equivalent to replicated, micronutrient-specific nutrigenomic evidence across cultured finfish species.

Micronutrients regulate transcription through redox balance, enzyme activity, membrane protection, immunity, endocrine signaling, and mineral metabolism. Their effects vary with chemical form, bioavailability, basal nutritional status, nutrient interactions, tissue, life stage, and supplementation level. Similar transcriptional responses may therefore reflect deficiency correction, adaptive compensation, or toxicity, depending on accompanying biochemical and phenotypic outcomes.

Vitamin E provides a dose–response example, altering hepatic and intestinal *nrf2*/*keap1*- and antioxidant-related transcripts [34]. As a lipid-soluble antioxidant, it limits membrane lipid peroxidation and may influence malondialdehyde, antioxidant enzymes, complement activity, immunoglobulin M, and *nrf2*/*keap1*-related transcription. Concordant molecular, biochemical, immune, and growth responses would support a relationship between vitamin E supply, redox regulation, and tissue health, whereas transcriptional changes alone do not confirm Nrf2/Keap1 pathway activation [4,10,34].

Micronutrient nutrigenomics should therefore be regarded as an emerging evidence area. Future studies should use graded, analytically verified micronutrient concentrations, and should distinguish deficiency correction from pharmacological supplementation. Coordinated measurements of tissue nutrient status, gene expression, protein abundance or activity, enzyme activity, oxidative damage, immunity, histology, growth, and survival are required before molecular responses can be translated into species-specific precision-formulation recommendations.

### 3.7. Carbohydrate and Systemic Energy Metabolism as an Emerging Area

Carbohydrate nutrigenomics remains one of the least developed areas within cultured-finfish precision nutrition. No dedicated series of controlled, graded carbohydrate-nutrigenomics studies was represented among the 21 feeding studies selected for detailed comparison. The available evidence instead arose indirectly from long-term plant-based diets and integrated phytobiotic–transcriptomic studies in which carbohydrate and energy-metabolism responses were embedded within broader changes in the protein source, lipid source, ingredient composition, or bioactive-compound exposure [14,39].

These studies identified responses involving glycolytic, gluconeogenic, lipid-energy, and endocrine pathways together with plasma metabolites, growth, and, in some cases, predicted microbial metabolic functions. However, such responses cannot be attributed specifically to dietary carbohydrate because the interventions simultaneously altered several nutritional or functional components. Predicted microbial carbohydrate metabolism also does not demonstrate substrate utilization or metabolite production without direct measurements of microbial activity and fermentation products.

Dedicated studies should therefore use isonitrogenous and isolipidic diets that vary digestible carbohydrate source and level while controlling processing, fiber, and energy density. Postprandial time-series sampling should integrate circulating glucose and related metabolites, hepatic and muscular glycogen, digestive and metabolic enzyme activities, glycolytic and gluconeogenic transcripts, metabolomics, tissue histology, microbiome function, growth, and feed utilization. Until such evidence is replicated across species with different feeding ecologies and glucose-regulatory capacities, carbohydrate and systemic energy nutrigenomics should be considered limited and emerging rather than an established precision aquafeed pathway.

### 3.8. Epigenetics, Nutritional Programming, and Environmental Stress Resilience

Nutritional programming should be distinguished from short-term nutritional modulation. Nutritional programming implies that parental, embryonic, larval, or early-life dietary exposure produces a response that persists after the original exposure or modifies the response to a later dietary or environmental challenge. Demonstrating such persistence requires clearly separated exposure and follow-up periods, appropriate later-life controls, and molecular or phenotypic outcomes measured after the programming diet has been removed or standardized.

Within the representative evidence reviewed here, direct nutritional programming evidence was provided principally by Türkmen et al. [15], who linked broodstock lipid composition and selection for high or low *fads2* expression with offspring *fads2*-promoter methylation, hepatic lipid-related expression, fatty-acid metabolism, and growth. The combined molecular and phenotypic findings suggest that parental lipid nutrition may have persistent consequences for offspring lipid metabolism. However, the complex broodstock–offspring design, small methylation subgroups, sample pooling, and absence of comparable independent studies across species mean that this evidence remains limited and emerging.

DNA methylation is only one potential mechanism of nutritional programming. Histone modification, chromatin accessibility, non-coding RNA regulation, and persistent changes in transcription-factor networks may also contribute to nutritional memory [10,13,35,36,37]. Nevertheless, these mechanisms were not directly validated in most of the representative finfish feeding studies. They should therefore be presented as candidate mechanisms and future research priorities rather than as established consequences of aquafeed manipulation.

Short-term pre-challenge nutritional interventions should also not be interpreted automatically as epigenetic programming. Ogun et al. [33] evaluated graded taurine supplementation in juvenile olive flounder subsequently exposed to acute and lethal temperature stress. Taurine influenced whole-body lipid, plasma-free amino acids, cortisol, and selected physiological responses, but had limited effects on antioxidant enzymes, immune parameters, heat-shock transcripts, and survival under severe thermal exposure. The expressions of *hsp60*, *hsp70*, *hsp90*, and *wap65* were not significantly altered by taurine during acute temperature stress [32,33]. This study therefore provides evidence of a diet–stress interaction rather than persistent or inherited epigenetic programming.

Future programming studies should include parental or early-life exposure, later-life follow-up under standardized diets, reciprocal or cross-over designs where feasible, multiple tissues, and direct assessment of DNA methylation, chromatin state, histone modifications, small RNAs, gene expression, metabolites, and functional phenotypes. Multigenerational and farm-relevant validation will be necessary to determine whether observed molecular marks are persistent, heritable, and practically useful for improving environmental resilience.

### 3.9. Cross-Cutting Pathway Synthesis

Apparent inconsistencies among studies should not necessarily be interpreted as failures of reproducibility because the reviewed experiments differ substantially in their biological, nutritional, and methodological contexts. For alternative proteins, juvenile yellow perch showed a relatively restricted intestinal transcriptomic response dominated by cholesterol biosynthesis, whereas Japanese seabass and groupers showed inflammatory, transporter, and enteritis-related responses following soybean-derived protein inclusion. These contrasting outcomes may reflect differences in species-specific tolerance, feeding ecology, developmental stage, ingredient processing, replacement level, amino-acid and bile-acid balance, feeding duration, intestinal region, and whether molecular responses were assessed before or after histological injury became evident [7,8,9,17,18,22].

Lipid-related responses are similarly context dependent. Increased expression of *fads2* or *elovl5* may indicate a transcriptional response consistent with increased demand for endogenous long-chain polyunsaturated fatty-acid biosynthesis in species with relatively high conversion capacities, whereas the same response may represent incomplete compensation in marine carnivorous species when tissue eicosapentaenoic acid and docosahexaenoic acid remain reduced. Differences in baseline fatty-acid status, dietary oil source, exposure duration, sampled tissue, developmental stage, and integration with lipidomic or tissue-composition outcomes can therefore result in different interpretations of similar molecular responses [3,14,15,16].

**Figure 2:**
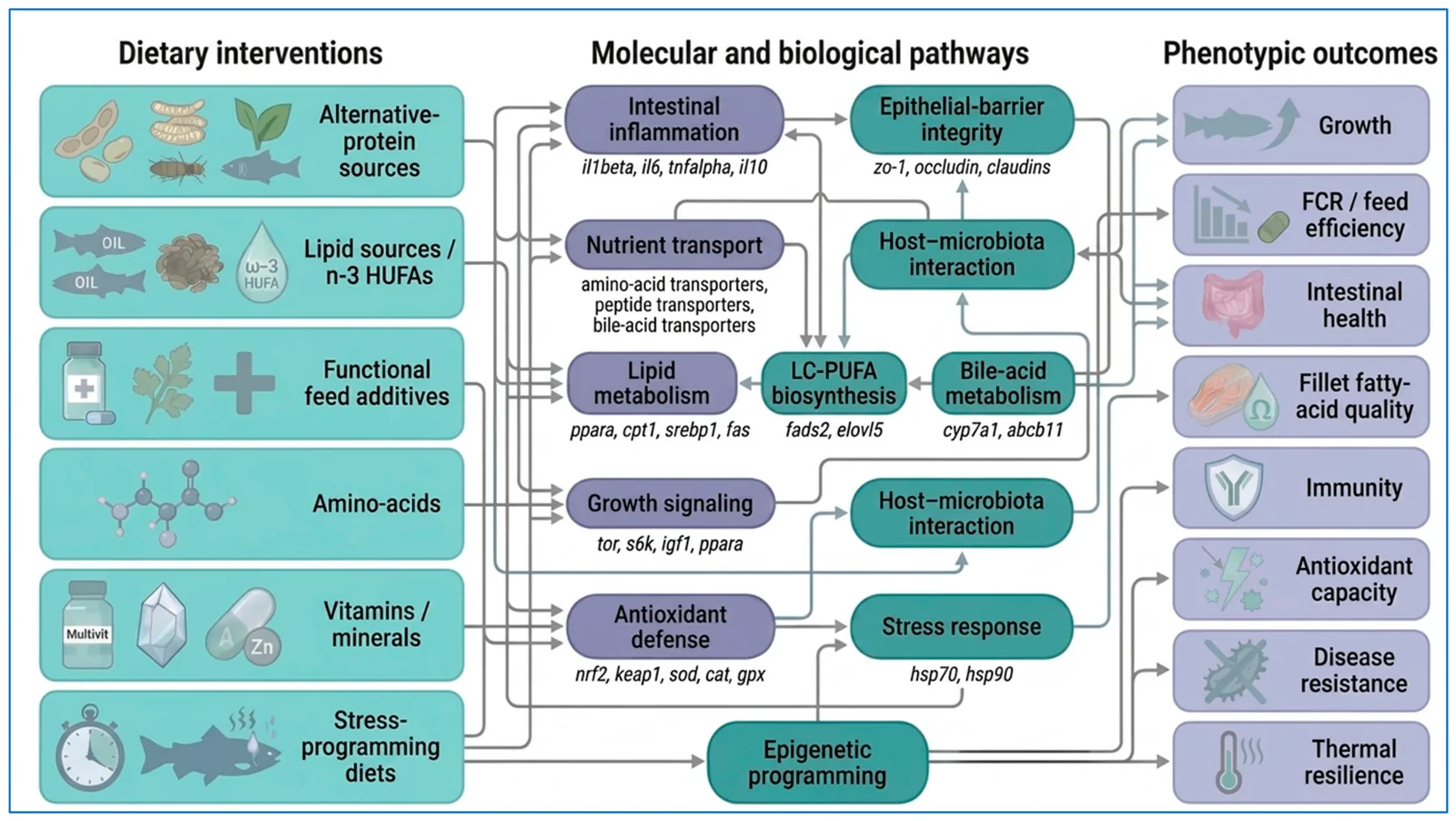
Diet-responsive pathway network linking dietary interventions, pathway-related molecular responses, and phenotypic outcomes in cultured finfish nutrigenomics. The network summarizes reported associations of alternative-protein sources, lipid sources and n-3 HUFAs, functional feed additives, amino acids, vitamins and micronutrients, and stress-programming diets with markers related to intestinal inflammation, epithelial-barrier integrity, nutrient transport, lipid metabolism, LC-PUFA biosynthesis, bile-acid metabolism, TOR/IGF signaling, Nrf2/Keap1 antioxidant defense, host–microbiota interactions, stress responses, and epigenetic or nutritional programming. The depicted connections represent an interpretive synthesis of evidence obtained across multiple studies; not every connection was demonstrated within a single experiment. The arrows therefore indicate proposed or reported associations and do not establish pathway activation, mediation, or causal direction. Molecular responses were considered together with growth, feed efficiency, intestinal health, fillet fatty-acid quality, immunity, antioxidant capacity, disease resistance, and thermal resilience.

Functional additives, amino acids, and stress-modulating diets introduce additional sources of variation. Probiotic and microbiota responses depend on strain identity, viable dose, basal diet, resident microbial community, host genotype, life stage, rearing environment, and challenge model, whereas methionine- and taurine-related responses depend on basal nutrient adequacy, supplementation level, target tissue, feeding duration, and stress severity [24,25,26,30,31,32,33,39,40,41]. Analytical differences, including targeted quantitative polymerase chain reaction versus RNA sequencing (RNA-seq), reference-gene validation, reference genome quality, differential-expression thresholds, sampling time, sample pooling, and tank-level replication, may further contribute to inconsistent findings. Cross-study interpretation should therefore prioritize agreement among biological pathways, tissue responses, and functional phenotypes rather than expecting identical regulation of individual genes across species and experimental conditions. The integrated relationships among dietary interventions, molecular and biological pathways, and their associated phenotypic outcomes are summarized in Figure 2.

Across the literature, alternative proteins, lipid-source manipulation, functional additives, amino acids, micronutrients, and stress-modulating diets were associated with intestinal inflammation, epithelial-barrier integrity, nutrient transport, lipid metabolism, long-chain polyunsaturated fatty-acid biosynthesis, bile-acid metabolism, TOR/IGF growth signaling, Nrf2/Keap1 antioxidant defense, host–microbiota interactions, stress responses, and nutritional programming. Table 2 summarizes the principal dietary evidence, directly measured outcomes, methodological limitations, and evidence level assigned to each pathway.

### 3.10. Methodological Challenges and Reporting Priorities

Interpretation across finfish nutrigenomics studies is constrained by variation in biological replication, statistical power, sample handling, molecular validation, sequencing depth, batch control, multiple-testing procedures, and data availability. The experimental unit must be distinguished clearly from fish-level subsampling and technical replication. In most feeding studies, the tank, cage, or independently managed culture unit is the biological replicate because fish sharing the same unit experience the same diet, water, microbial environment, and husbandry conditions. Multiple fish sampled from one tank can characterize within-tank variation but do not increase the number of independent experimental units. Treating individual fish from the same tank as independent replicates can therefore produce pseudoreplication, underestimate uncertainty, and inflate apparent statistical significance [42].

Statistical power depends primarily on the number of independent experimental units, the expected treatment effect, and between-unit biological variation. Sample-size justification should therefore be based on the tank- or cage-level analysis rather than the total number of fish stocked or sampled. Nested or mixed-effects models are appropriate when several fish, tissues, or molecular measurements are collected from each experimental unit. In sequencing studies, greater read depth can improve transcript detection and estimation precision, but it cannot compensate for inadequate biological replication. A small number of deeply sequenced libraries may provide less reliable evidence of treatment effects than a larger number of adequately sequenced independent biological libraries [10,12,13].

Quantitative polymerase chain reaction requires validation at both the assay and normalization levels. Primer specificity, amplification efficiency, RNA integrity, reverse-transcription procedures, negative controls, and the number of biological and technical replicates should be reported according to the Minimum Information for Publication of Quantitative Real-Time PCR Experiments consistent principles [38]. Reference genes should be evaluated for stability across the actual diets, tissues, developmental stages, and stress conditions under investigation, preferably using multiple candidate genes and an appropriate stability-assessment procedure. Reliance on a single unvalidated housekeeping gene can create or obscure apparent treatment-related fold changes. Quantitative polymerase chain reaction confirmation of RNA sequencing results using the same RNA samples provides valuable cross-platform confirmation, but it should not be described as independent biological validation. Stronger validation uses independent biological samples, a separate experimental cohort, or complementary protein, metabolite, enzyme-activity, histological, or physiological measurements.

RNA sequencing reliability depends on RNA quality, independent library number, library-preparation procedures, sequencing depth, read quality, mapping rate, library complexity, reference-genome or transcriptome quality, and the statistical model used for differential-expression analysis. Treatment groups should be balanced across RNA extraction dates, library-preparation batches, sequencing lanes, and sequencing runs so that technical batch is not confounded with diet. Potential batch effects and outlying libraries should be evaluated using sample-correlation analysis, dimensionality-reduction approaches, and other quality-control procedures, and known batch variables should be incorporated into the differential-expression model when appropriate. Read depth should therefore be interpreted together with biological replication, mapping quality, transcript coverage, and library complexity rather than as an isolated indicator of study quality.

Multiple-testing correction is essential because transcriptomic, microbiome, metabolomic, and pathway-enrichment analyses evaluate large numbers of features simultaneously. Reliance on unadjusted *p*-values can generate substantial numbers of false-positive genes, taxa, metabolites, or pathways. Studies should report the normalization method, statistical model, fold-change criterion, raw and adjusted probability thresholds, false discovery rate procedure, pathway database and version, enrichment background, and treatment of low-abundance features. Selected RNA sequencing results should be confirmed using validated quantitative polymerase chain reaction assays when possible, while pathway-level conclusions should be supported by concordant expression of multiple pathway components and relevant protein, metabolite, biochemical, histological, or phenotypic outcomes.

Reproducibility also requires transparent access to the information needed to repeat the analysis. Raw sequence reads, processed count matrices, quantitative polymerase chain reaction cycle-threshold data where feasible, primer information, sample metadata, analysis code, software versions, reference-genome or transcriptome versions, statistical parameters, and data-accession numbers should be reported or deposited in appropriate repositories. Technical confirmation, biological replication, and external validation should be distinguished explicitly. Supplementary Table S2 summarizes the molecular platforms, library construction or pooling, sequencing scale and quality, statistical criteria, and public data-accession status of the representative studies and identifies information that was not reported. Evidence was interpreted more cautiously when biological replication, normalization procedures, batch handling, multiple-testing correction, or independent validation could not be established. The principal methodological and reporting priorities are summarized in Table 3.

## 4. Microorganisms at the Diet–Host Interface

### 4.1. Fish-Associated Microbial Niches and Ecological Context

A fish microbiome is not a single intestinal community. Microorganisms occupy the digesta, mucus layer, epithelial surface, gills, skin, feed, rearing water, sediments, and tank biofilms. These compartments exchange organisms at different rates and together form a dynamic host-associated microbial ecosystem.

Nutritional studies usually focus on the intestine because it is the principal site of feed–microbe–host interaction. However, luminal samples can overrepresent transient microorganisms derived from feed or rearing water. Mucosa-associated communities are more likely to interact directly with epithelial receptors and immune cells, although they are more difficult to sample consistently [27,28].

The ecological meaning of a microbial shift therefore depends strongly on sampling location and experimental context. A taxon enriched in digesta may reflect recent ingestion rather than stable colonization, whereas a low-abundance mucosal organism may exert disproportionate effects on epithelial or immune processes.

Sampling procedures can also influence the observed microbial profile. Fasted versus postprandial sampling, anterior versus distal intestine, pooled versus individual samples, and rinsed versus unrinsed tissues may produce different community patterns. Feed, source water, and tank biofilms should therefore be sampled concurrently to distinguish diet-associated inoculation from host selection and environmental transmission [27,29].

Bacteria dominate current evidence because 16S rRNA amplicon sequencing is accessible and well established. Nevertheless, the fish microbiome also includes archaea, fungi, protists, viruses, and bacteriophages that may influence fermentation, ingredient degradation, and opportunistic bacteria. Accordingly, microbiome refers to the community together with its genes and functions, whereas microbiota refers primarily to the organisms detected.

### 4.2. Dietary Substrates, Microbial Succession, and Dysbiosis

Diet is both a nutrient source for fish and an ecological filter for microorganisms. Protein source, carbohydrate complexity, lipid class, fiber, chitin, antinutritional factors, bile-acid availability, micronutrients, and feed processing determine which substrates reach the distal intestine.

Alternative proteins can increase the availability of fermentable polysaccharides or resistant proteins, whereas fish oil replacement and taurine status can alter bile-acid pools with antimicrobial and signaling functions. Therefore, identical inclusion levels may produce different microbial outcomes depending on digestibility, processing, fish species, and background formulation.

Soybean-derived feeds also illustrate how enteritis can coincide with microbial restructuring in sensitive carnivorous fish. Because the associated host inflammatory and barrier responses are discussed in Section 3.2, the main microbiome implication is that ingredient processing, substrate availability, and mucosal condition may alter community succession [8,9,21,22].

These changes should not be interpreted using a simple “high diversity is healthy” rule. Microbial richness may increase during ecological instability, whereas a lower-diversity community may remain functional and resilient. Health assessment should therefore prioritize functional capacity, temporal stability, mucosal localization, and agreement with histological and host-response data.

Naya-Catala et al. [25] showed that intestinal microbiota succession in gilthead sea bream was jointly shaped by diet, host genetic background, and production stage. Piazzon et al. [24] likewise demonstrated that a fishmeal-free formulation could support growth while reshaping the microbiota and tissue-specific gene expression without increasing intestinal parasite susceptibility. These findings indicate that microbial responses are context dependent and may vary with genotype, season, salinity, life stage, and baseline microbial composition.

### 4.3. Live Microbial Interventions, Fermented Ingredients, and Microbial Biomass

Live microbial interventions should be distinguished from fermented ingredients and microbial biomass. Probiotics and synbiotics depend on the delivery of viable, strain-defined microorganisms and may act through digestive-enzyme secretion, organic-acid or bacteriocin production, competition for nutrients and adhesion sites, modification of mucus and tight junctions, immune regulation, and restructuring of resident microbial communities. In contrast, fermented ingredients and microbial biomass may act through altered substrate composition, residual metabolites, peptides, nucleotides, enzymes, or microbial cell-wall components, with or without viable cells.

The microorganism delivered through feed does not need to become a permanent intestinal resident to exert an effect. Transient passage may alter the intestinal chemical environment or host signaling. Therefore, failure to detect long-term colonization does not necessarily indicate ineffectiveness, while detection alone does not demonstrate biological activity.

The grouper and gray mullet studies summarized above illustrate why strain identity is important. Xie et al. [40] showed that single- and mixed-strain preparations produced different effects on growth, digestive enzymes, antioxidant responses, immune-gene expression, and microbial community structure. Chan et al. [41] likewise reported strain-specific differences in pathogen antagonism, immune activation, microbial remodeling, and protection against *N. seriolae*.

A credible probiotic claim should therefore include strain-level identification, viable counts, feed stability, host dose, treatment duration, target pathogen, and at least one mechanistic endpoint. Reliance on improved growth alone is insufficient because similar growth responses may result from different microbial or host mechanisms.

Fermented ingredients and microbial biomass extend this concept beyond live probiotics. Fermentation can reduce antigenic proteins and antinutritional factors while releasing peptides, organic acids, and microbial cell-wall components. Yeasts, bacteria, fungi, and microalgae may also serve as single-cell proteins, nucleotide sources, enzyme carriers, or immunologically active biomass. Their nutritional contribution should be distinguished from microbial bioactivity by characterizing the production organism, residual viability, metabolite profile, cell-wall composition, digestibility, and batch consistency.

### 4.4. Microbial Metabolites as Nutrigenomic Signals

Microbial metabolites provide a plausible explanatory link between diet composition and host transcription, but this relationship remains inferential unless metabolite production, host exposure, and downstream responses are measured directly. Short-chain fatty acids such as acetate, propionate, and butyrate can arise from microbial fermentation of available carbohydrates, while lactate may support cross-feeding among intestinal microorganisms.

In fish, butyrate-generating strategies and tributyrin supplementation have been associated with intestinal morphology, barrier-gene expression, inflammatory tone, and microbial restructuring [26]. Butyrate may also serve as an epithelial energy source and influence histone deacetylation, tight-junction regulation, and immune signaling, although these mechanisms require species-specific validation.

Microorganisms can also modify amino acids, vitamins, and bile acids. Microbial amino-acid metabolism may generate indoles, amines, sulfur compounds, and other signaling molecules, whereas bile-acid transformation can alter antimicrobial pressure and receptor activation.

These processes are particularly relevant when plant-based diets modify taurine availability, lipid digestion, and enterohepatic cycling. However, most finfish studies infer microbial function from taxonomic composition or predicted pathways rather than directly measuring metabolite flux. Targeted or untargeted metabolomics should therefore accompany microbial profiling when metabolite-mediated mechanisms are proposed.

The tilapia study by Chen et al. [39] illustrates both the potential and limitations of integrated inference. Overlap between predicted microbial pathways and host transcriptomic responses can identify candidate diet–microbe–host relationships, but it does not confirm that the microorganisms produced the relevant metabolites. Stronger evidence requires absolute microbial quantification, direct metabolite measurements, cultured isolates or synthetic communities, and experimental validation of the microbial contribution.

### 4.5. Pathobionts, Pathogens, Disease Resistance, and Microbial Safety

A pathobiont is a resident or recurrent microorganism that can become harmful when host defenses, microbial competition, or environmental conditions change. Diet-induced inflammation, hypoxia, high temperature, crowding, and epithelial-barrier damage can increase the likelihood that opportunistic microorganisms express virulence or invade host tissues.

An increase in a genus containing pathogenic members is therefore not equivalent to disease, and a decrease in relative abundance does not prove protection. Pathogenic interpretation requires strain-level identity, absolute microbial load, virulence genes, microbial activity, tissue invasion, and corresponding host pathology.

Challenge models provide stronger functional evidence than community profiling alone. In gray mullet, *L. rhamnosus* supplementation altered immune transcripts and the intestinal microbial community while improving survival after *N. seriolae* challenge [41].

Reductions in potential pathobionts such as *Mycoplasma* have also accompanied improved host responses [40]. However, genus-level associations should be interpreted cautiously because the same taxon may contain commensal, neutral, and harmful strains. Future studies should therefore measure pathogen burden and challenge-relevant virulence markers rather than relying only on survival.

Microbial feed additives also require biosafety assessment, particularly when candidate strains belong or are closely related to the *Bacillus cereus* group. Safety evaluation should include strain-level or genome-resolved identification; screening for toxin genes, virulence factors, and transferable antimicrobial-resistance genes; and assessment of genetic stability and undesirable metabolite production. Manufacturing should verify strain purity, viable dose, feed and storage stability, batch consistency, and absence of contamination. Improved growth, antioxidant responses, or immune-gene expression alone should not be considered evidence of probiotic safety.

### 4.6. From Association to Causality: Microbiome Methods

Most fish nutritional microbiome studies use 16S rRNA amplicon sequencing. This approach is useful for comparing microbial communities, but it is compositional, generally reports relative rather than absolute abundance, may not resolve strains, and does not establish microbial viability or metabolic activity.

Functional predictions based on marker genes should therefore be described as inferred rather than directly measured. Quantitative PCR, flow cytometry, spike-in standards, and digital PCR can provide estimates of absolute microbial load, whereas shotgun metagenomics can improve taxonomic and functional resolution.

Metatranscriptomics identifies actively expressed microbial genes, metabolomics measures chemical outputs, and culturomics recovers isolates for mechanistic testing. Stable-isotope tracing can further connect dietary substrates with microbial transformation and subsequent host metabolite production.

Causal relationships may be examined using gnotobiotic fish, carefully controlled antibiotic perturbation, microbial transplantation, or defined synthetic communities. Although these approaches have ecological and animal-welfare limitations, combining them with host RNA-seq can help determine whether microbial functions can precede and predict epithelial, immune, hepatic, or muscular transcriptional responses [28,29].

Microbiome experiments require treatment-level replication and appropriate contamination controls because the tank functions as both an ecological and statistical unit. These general design, quality-control, and reporting requirements are summarized in Section 3.10 and Table 3. The microbiome-specific priority is to distinguish environmental transmission and transient feed-associated organisms from resident mucosal communities and metabolically active microorganisms.

### 4.7. Integrating Microbial and Host Omics for Precision Aquafeeds

An informative precision nutrition framework is a proposed sequence linking diet, microorganisms, host responses, and production outcomes. In most reviewed studies, these components were measured within the same feeding trial; therefore, their covariation identifies candidate mechanisms but does not establish that one component caused the next.

Diet composition may alter the substrates and inocula available to the intestinal ecosystem, while microbial taxa, genes, or metabolites may covary with host transcription and physiological outcomes. However, taxonomic shifts, predicted microbial functions, and correlated host responses do not demonstrate microbial mediation.

Integrated diet–microbiome–host studies should therefore prioritize temporal ordering and causal testing rather than simply increasing the number of analytical platforms. Longitudinal sampling, absolute microbial quantification, direct metabolite measurement, and targeted microbial perturbation can help determine whether microbial responses precede and contribute to host adaptation. The analytical approach should be selected according to the proposed mechanism rather than applying every omics platform to every feeding trial. The major microorganism-centered mechanisms are summarized in Table 4.

## 5. Integrative Mechanistic Frameworks

### 5.1. From Molecular Response to Precision Aquafeed Function

A central contribution of nutrigenomics is its ability to explain why diets with similar short-term growth performance may produce different long-term intestinal, metabolic, immune, or stress-resilience outcomes. This distinction is important because conventional production indicators may not reveal subclinical or tissue-specific effects.

Across representative studies, the most informative domains include intestinal inflammation and barrier integrity, lipid metabolism and LC-PUFA biosynthesis, amino-acid transport and TOR/IGF signaling, antioxidant regulation, host–microbiota interactions, and stress physiology. These pathways provide an interpretive framework for evaluating dietary responses, although transcriptional evidence alone does not confirm corresponding protein-level or physiological changes.

This evidence supports a precision aquafeed approach in which feed suitability is assessed not only through weight gain and feed conversion but also through tissue-specific molecular adaptation. Molecular responses should therefore be interpreted together with physiological and production-level outcomes.

The most extensively represented evidence concerns alternative proteins and lipid-source manipulation. Their effects are synthesized below through the gut–liver–muscle and lipid–bile-acid frameworks, with emphasis on coordinated pathway and phenotypic responses.

### 5.2. Gut–Liver–Muscle Axis

Alternative-protein nutrigenomics should be interpreted through a gut–liver–muscle framework. The intestine responds directly to dietary antinutritional factors, microbial metabolites, altered bile acids, and nutrient availability. The liver integrates amino-acid, lipid, detoxification, bile-acid, and energy metabolism, whereas muscle reflects protein accretion, flesh quality, and myogenic signaling.

**Figure 3:**
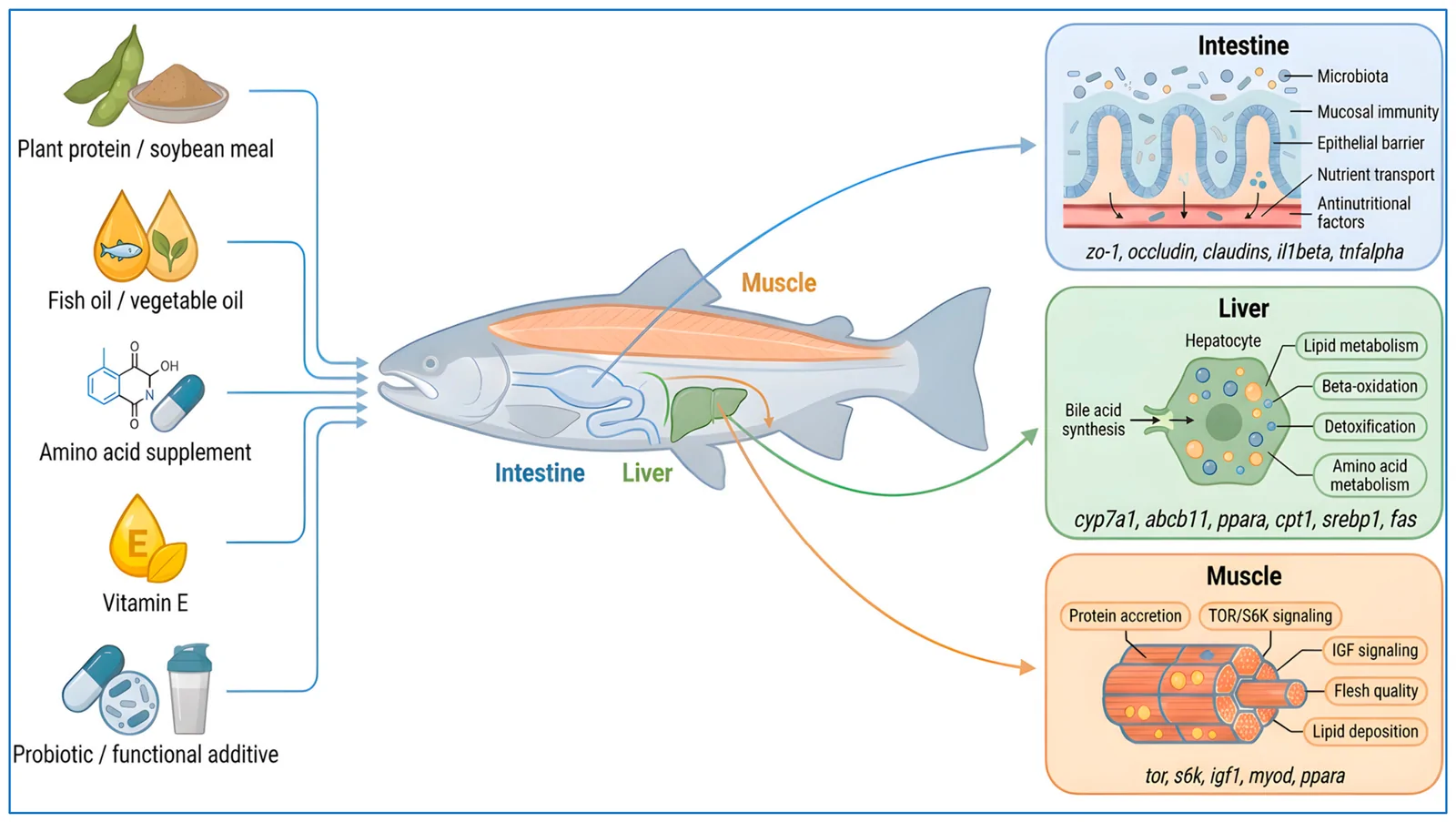
Proposed gut–liver–muscle framework for interpreting precision finfish nutrigenomics. Dietary ingredients and functional nutrients first interact with the intestine, where antinutritional factors, microbiota, mucosal immunity, epithelial-barrier integrity, and nutrient transport influence feed tolerance. The liver integrates lipid metabolism, bile-acid synthesis, β-oxidation, detoxification, and amino-acid metabolism through pathways involving genes such as *cyp7a1*, *abcb11*, *ppara*, *cpt1*, *srebp1*, and *fas*. Muscle reflects responses related to protein accretion, TOR/S6K and IGF signaling, flesh quality, myogenic regulation, and lipid deposition, including changes in *tor*, *s6k*, *igf1*, *myod*, and *myog*. The arrows represent proposed tissue-level relationships synthesized from evidence obtained across different studies and do not indicate that direct signaling among the intestine, liver, and muscle was demonstrated experimentally. The framework does not establish pathway activation or causal direction.

Alternative-protein studies summarized in Section 3.2 show that intestinal tolerance varies with ingredient type, processing, inclusion level, and species [7,8,9,17,18,22]. These tissue-specific responses are summarized within a proposed gut–liver–muscle framework that organizes associations among intestinal condition, hepatic regulation, muscle growth, lipid deposition, and flesh quality (Figure 3). This framework is interpretive and does not imply that the reviewed studies directly demonstrated causal signaling among these tissues.

Soybean-derived proteins may disturb intestinal homeostasis through inflammatory activation, epithelial-barrier impairment, and altered nutrient transport, particularly at excessive inclusion levels. These responses should be evaluated together with intestinal histology, microbiota, growth, and feed efficiency rather than through individual molecular markers alone. Fermentation or ingredient refinement may reduce antinutritional pressure but does not eliminate dose- and species-dependent intolerance, whereas other novel protein sources may exert distinct effects through chitin, microbial cell-wall components, nucleotides, peptides, pigments, and lipid fractions [8,9,13,17,18,19,20]. The current gut–liver–muscle interpretation is therefore supported mainly by soybean-based studies and should not be generalized directly to insect, microbial, single-cell, or microalgal proteins until comparable multi-tissue and phenotype-integrated evidence becomes available.

### 5.3. Lipid and Bile-Acid Integration

Lipid nutrigenomics is central to precision aquafeeds because fish oil replacement can affect energy metabolism, tissue fatty-acid composition, fillet nutritional value, inflammatory balance, reproductive performance, and stress physiology. These responses should be interpreted together with dietary fatty-acid profiles, tissue EPA and DHA retention, hepatic lipid accumulation, bile-acid and other relevant metabolite concentrations, protein abundance or enzyme activity when available, oxidative markers, and growth performance [3,14,15,16,32].

The main molecular responses can be organized into three modules: lipid catabolism, involving *ppara*, *cpt1*, and β-oxidation-related genes; lipogenesis and lipid deposition, involving *srebp1*, *fas*, and triacylglycerol-related genes; and LC-PUFA biosynthesis, involving *fads2* and *elovl5*. This pathway-based framework helps connect transcriptional responses with lipid utilization and tissue quality.

Upregulation of *fads2* and *elovl5* is often interpreted as a compensatory transcriptional response to reduced dietary EPA and DHA rather than as direct evidence that long-chain polyunsaturated fatty-acid biosynthetic activity increased. However, the effectiveness of this response differs among species, with freshwater and omnivorous fish generally showing greater biosynthetic flexibility than marine carnivores.

Therefore, increased *fads2* expression does not necessarily indicate full restoration of tissue EPA and DHA. It may instead reflect an incomplete compensatory response that remains insufficient at the phenotypic level. Gene-expression results should therefore be evaluated together with tissue fatty-acid composition and lipid-retention data [3,15,16].

**Figure 4:**
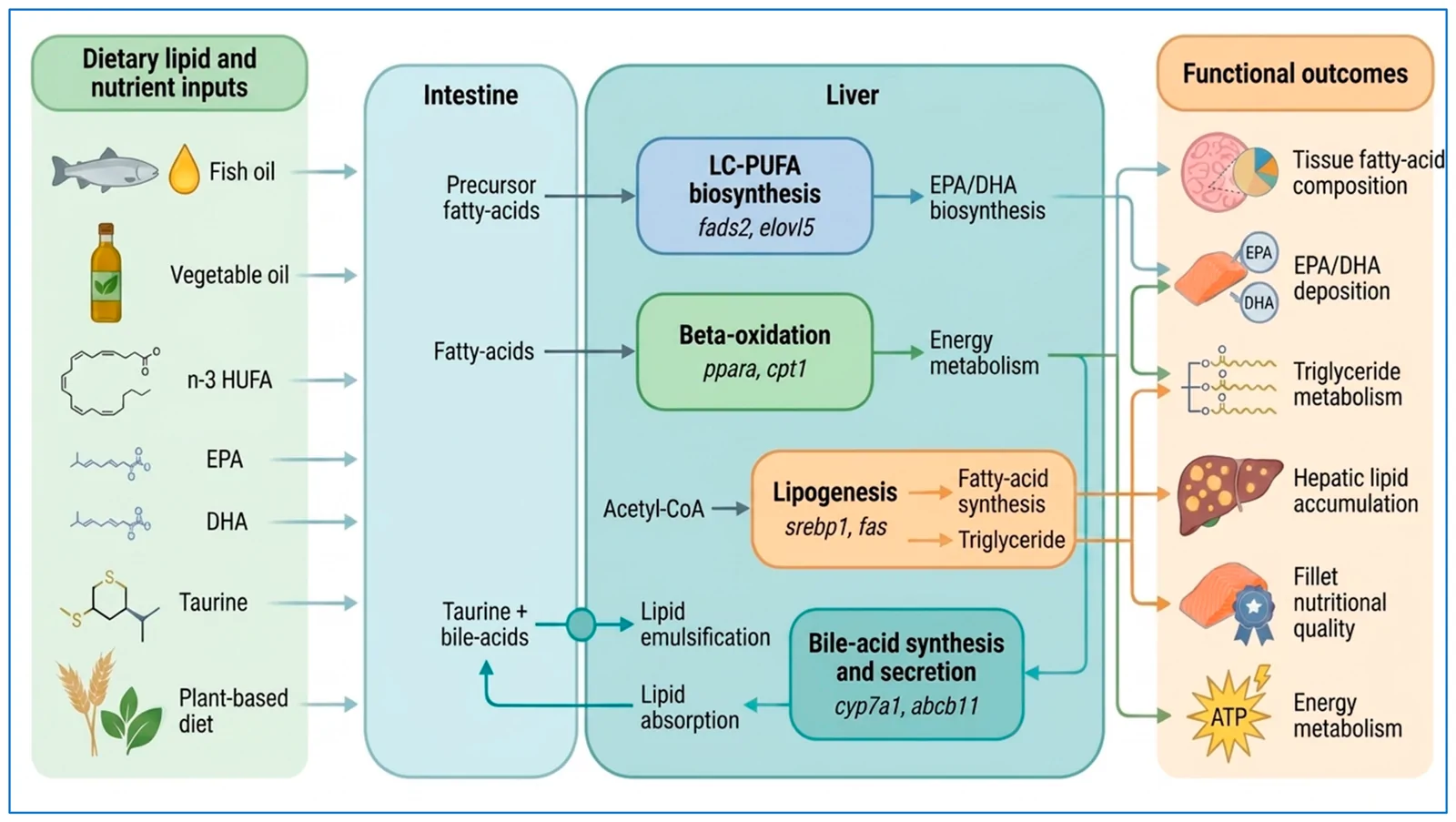
Interpretive framework for lipid and bile-acid nutrigenomics in cultured finfish. Dietary lipid inputs, including fish oil, vegetable oil, n-3 highly unsaturated fatty acids (n-3 HUFAs), eicosapentaenoic acid (EPA), docosahexaenoic acid (DHA), taurine, and plant-based diets, were associated with transcriptomic, biochemical, and tissue-composition responses related to lipid metabolism. Long-chain polyunsaturated fatty-acid biosynthesis-related markers include *fads2* and *elovl5*; β-oxidation-related markers include *ppara* and *cpt1*; lipogenesis-related markers include *srebp1* and *fas*; and bile-acid synthesis- and secretion-related markers include *cyp7a1* and *abcb11*. Taurine supplementation was associated with bile-acid and lipid-utilization outcomes in fish fed plant-based diets. The diagram summarizes reported associations and does not demonstrate pathway activity or causal direction among the depicted components.

Aragão et al. [32] showed that taurine supplementation improved bile-acid- and lipid-related physiological outcomes in Senegalese sole fed plant-based diets. At the transcript level, 0.5% taurine increased *cyp7a1* and *abcb11* relative to the non-supplemented plant-protein diet, although full restoration to the fishmeal expression level was not demonstrated. Precision lipid formulation should therefore consider oil source, fatty-acid profile, taurine availability, bile-acid metabolism, lipid digestion, and hepatic export capacity. These integrated mechanisms are summarized in Figure 4 [32,33].

This pathway-level interpretation emphasizes that lipid nutrigenomics should be evaluated together with tissue fatty-acid composition, lipid class distribution, bile-acid metabolism, hepatic lipid status, and growth or feed-efficiency outcomes.

### 5.4. Functional Additives, Microbiota, and Microbial Metabolites

Functional-additive responses were highly context dependent. In the reviewed studies, their direction and magnitude varied with additive category, microbial strain, viable dose, basal diet, resident microbiota, host genotype, life stage, rearing environment, and challenge model [24,25,26,39,40,41]. Increased immune-gene expression was not uniformly beneficial; its interpretation depended on whether it coincided with improved epithelial integrity, lower oxidative injury, growth, or challenge survival.

Functional-additive mechanisms were grouped as live microbial interventions, including probiotics and synbiotics; microbiota-directed substrates, including prebiotics; metabolite- or postbiotic-based strategies, including tributyrin and organic acids; plant-derived bioactives or phytobiotics; and fermentation-derived ingredients or microbial biomass. Although these categories may converge on epithelial integrity, immune balance, microbial ecology, and pathogen resistance, they should not be interpreted as mechanistically equivalent [23,24,25,26,39,40,41].

The reviewed probiotic studies also produced contrasting outcomes. Mixed microbial preparations in hybrid grouper were associated with favorable digestive, antioxidant, immune, and microbial responses [40], whereas strain-specific effects in gray mullet differed across growth, immune, microbiome, and *N. seriolae* challenge outcomes [41]. In gilthead sea bream, a fishmeal-free functional formulation altered microbiota and tissue-specific transcription without increasing parasite susceptibility [24]. These findings show that similar microbial or immune responses cannot be generalized across strains, host species, dietary backgrounds, or challenge models.

Immune activation should not automatically be interpreted as beneficial. Strong increases in *il1beta*, *il6*, *tnfalpha*, *tlr*, *myd88*, or NF-κB may indicate damaging inflammation when accompanied by intestinal lesions, poor growth, oxidative injury, or impaired barrier-gene expression.

Conversely, balanced immune readiness, improved mucosal barrier markers, reduced oxidative damage, and stronger disease resistance support a protective interpretation. Gene-expression changes should therefore be evaluated within the tissue context, challenge condition, and associated phenotype rather than assuming that upregulation indicates improvement [4,23,24,26].

Host genetics may further modify dietary and additive responses. Naya-Catala et al. [25] showed that genetics and nutrition influence intestinal microbiota succession and host–transcriptome interactions in gilthead sea bream. Functional feeds may therefore require genotype-aware or stock-specific validation because responses can vary with life stage, basal diet, microbiota, production cycle, water temperature, pathogen exposure, and stress history [13,24,25].

### 5.5. Amino Acids, Micronutrients, and Stress Resilience

Amino-acid responses depended strongly on basal adequacy, dose, species, and measured endpoint. In juvenile cobia, graded methionine supplementation produced a nonlinear response, with optimal growth and selected TOR/IGF-related outcomes occurring near an intermediate dietary concentration rather than increasing continuously with dose [30]. In largemouth bass, comparison of adequate and deficient diets showed coordinated growth, amino-acid, transporter, and TOR-pathway responses [31]. These studies support the importance of methionine adequacy but do not establish one transferable dose or molecular threshold across species.

Taurine outcomes were similarly context dependent. In Senegalese sole fed plant-based diets, taurine supplementation improved tissue taurine status and several bile-acid- and lipid-utilization outcomes, with partial recovery of related gene-expression responses [32]. In contrast, graded taurine supplementation in olive flounder altered selected lipid and stress-related physiological indices but did not substantially modify heat-shock transcripts or improve survival during lethal thermal exposure [33]. The difference may reflect species, basal formulation, target function, exposure duration, and the severity of the subsequent stress rather than a simple contradiction in taurine efficacy.

Micronutrient responses also followed endpoint-dependent dose patterns. The vitamin E study showed dose-related changes in growth, immunity, antioxidant enzymes, oxidative-damage markers, and *nrf2*/*keap1*-related transcription, but the estimated optimum depended on the endpoint considered [34]. Correction of deficiency, achievement of adequacy, and excessive supplementation may therefore produce different molecular and phenotypic responses. Species-specific dose–response studies are required before transcript-based thresholds can be translated into practical formulation recommendations.

Collectively, these studies demonstrate that molecular compensation does not necessarily indicate improved functional resilience. Nutrient effects should be interpreted according to basal nutritional status, dose, life stage, species, tissue, exposure duration, and the severity of the physiological or environmental challenge.

### 5.6. Phenotype Alignment and Biological Interpretation

Biological interpretation is most robust when molecular and phenotypic outcomes agree. Altered messenger RNA abundance should not be interpreted automatically as a corresponding change in protein abundance, enzyme activity, or physiological function. Post-transcriptional regulation, translation efficiency, protein degradation, post-translational modification, and tissue-specific protein localization can produce substantial divergence between transcript and protein responses. Accordingly, quantitative polymerase chain reaction and RNA-seq findings should primarily be regarded as evidence of transcriptional regulation or potential pathway involvement unless they are supported by protein-level, biochemical, metabolic, histological, or physiological validation. Strong functional inference therefore requires concordance among messenger RNA expression, protein abundance or activity, downstream metabolites, and relevant phenotypic outcomes [10,11,12,13,43]. Where such supporting evidence was unavailable in the reviewed studies, conclusions were restricted to transcriptional associations or potential pathway involvement rather than confirmation of biological function. Increased transcript abundance of *TOR*, *S6K*, *ASCT2*, and *IGF-I* is most informative when accompanied by improved growth, feed efficiency, protein retention, or muscle accretion [30,31]. Regulation of *nrf2*/*keap1*-related antioxidant genes is strengthened by concordant antioxidant enzyme activity and lower oxidative damage [34].

Changes in cytokines, tight-junction genes, and mucins are most biologically informative when linked with intestinal histology, enteritis scores, microbiota, growth, or challenge outcomes [9,17,24,26]. Conversely, transcriptional compensation without phenotypic recovery should be interpreted as an attempted adaptation rather than proof of nutritional adequacy. To provide an accessible synthesis of the evidence reviewed in Section 3, Section 4 and Section 5, the major dietary interventions, key molecular pathways, principal microbiome responses, and practical implications for aquaculture are summarized in Table 5.

## 6. Key Knowledge Gaps and Priority Research Directions

This review was designed as a structured narrative synthesis to provide a focused, pathway-centered interpretation of nutrigenomic and diet–microbiome–host evidence in cultured finfish. The literature search covered multiple databases and incorporated backward and forward citation tracking, allowing representative and mechanistically informative studies to be examined across major dietary themes. A total of 43 publications were retained in the overall synthesis, including 21 controlled feeding studies selected for detailed comparison. Because the review addressed a broad and rapidly developing field, some dietary interventions, fish species, life stages, and analytical approaches were represented by relatively few studies. In addition, substantial variation in species, feeding ecology, developmental stage, diet formulation, treatment dose, feeding duration, experimental unit, tissue, analytical platform, and outcome definition limited direct quantitative comparison across studies. A formal study-quality or risk-of-bias assessment was not undertaken; instead, the evidence levels used in this review were applied as descriptive synthesis categories to support transparent interpretation. Statistical pooling was also not performed because comparable variance estimates, endpoint definitions, and independent experimental-unit sample sizes were not consistently available. Accordingly, the present review provides a structured and transparent synthesis of representative evidence while identifying areas in which broader and more standardized research will further strengthen precision aquafeed development. Detailed within-study quantitative outcomes are provided in Supplementary Table S1, whereas molecular platforms, library construction or pooling, sequencing characteristics, statistical criteria, and data-accession status are summarized in Supplementary Table S2. These values were not statistically pooled because the studies differed substantially in species, developmental stage, diet composition, treatment dose, feeding duration, experimental unit, tissue, analytical platform, and outcome definition.

Alternative-protein and lipid studies are comparatively well-represented, whereas micronutrient nutrigenomics, epigenetic programming, microbial functional analysis, and realistic environmental-challenge studies remain less developed. Within the alternative-protein literature, the evidence remains strongly concentrated on soybean-derived ingredients, whereas insect meals, microbial and single-cell proteins, and microalgae have received substantially less nutrigenomic, multi-tissue, and microbiome-integrated investigation. Much of the available evidence is also associative and does not establish causal relationships among dietary exposure, microbial activity, host transcription, and functional phenotype.

A major priority is to move beyond short, targeted gene panels toward longitudinal and multi-tissue designs that better resolve cause and consequence. Studies should distinguish adaptive from pathological transcription, determine whether early molecular responses persist, and assess whether these signatures predict performance under commercial variation in temperature, density, water quality, pathogen exposure, and feed processing.

Greater use of metagenomics, metatranscriptomics, metabolomics, lipidomics, proteomics, and epigenomics will be necessary to connect microbial functions and host regulation with measurable nutrient fluxes and production outcomes. Single-cell RNA sequencing can identify cell-type-specific dietary responses that are masked in bulk-tissue analyses, while spatial transcriptomics can retain the anatomical context of intestinal, hepatic, muscular, and immune responses. Long-read sequencing can improve transcript isoform identification, splice-variant resolution, and genome or transcriptome annotation, particularly in non-model finfish species. Epigenomic approaches can further identify DNA methylation, chromatin accessibility, and other regulatory changes associated with nutritional programming [10,11,12,13,35,36,37].

**Figure 5:**
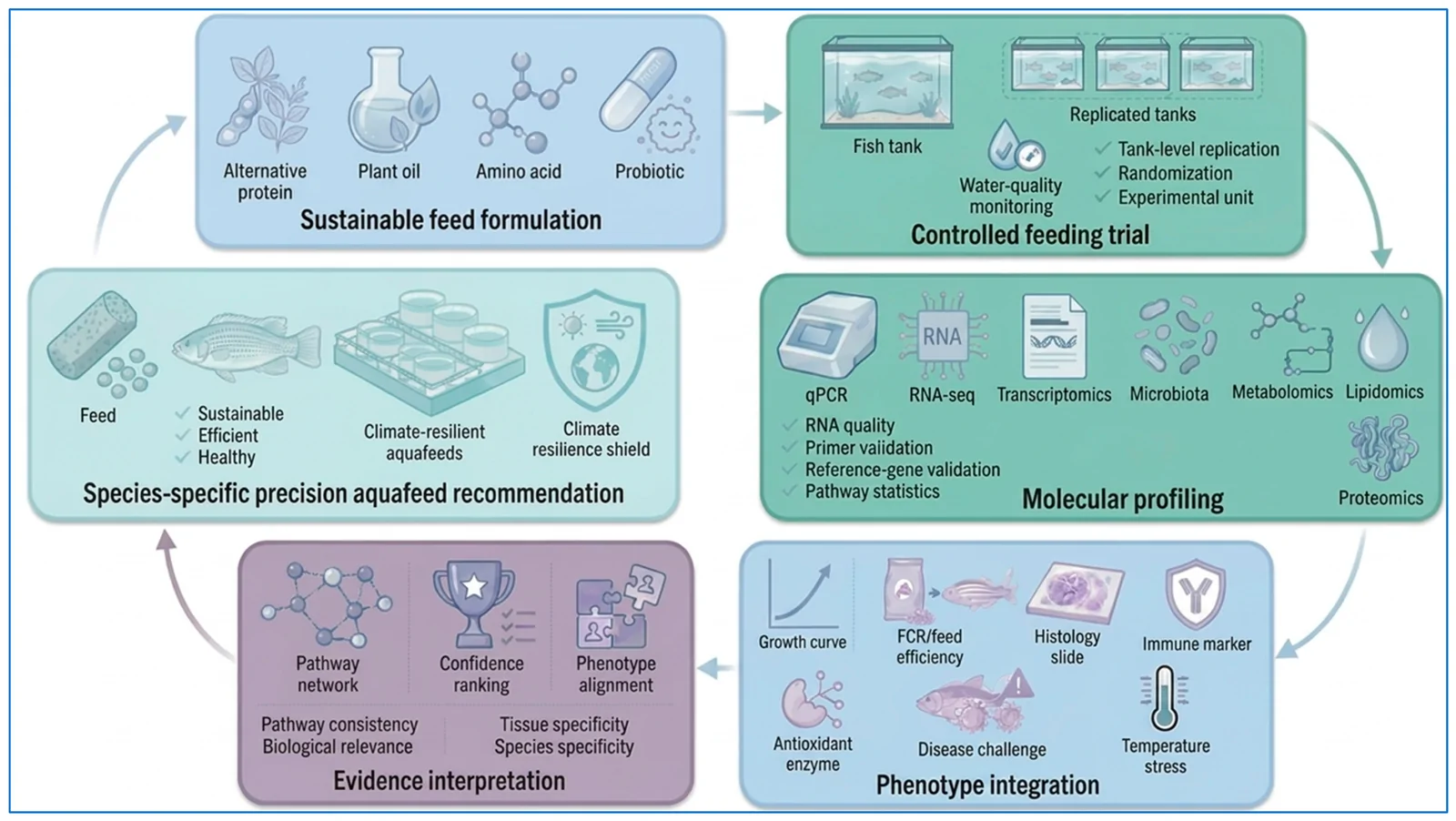
Future framework for precision aquafeed nutrigenomics. The proposed workflow begins with sustainable feed formulation using alternative proteins, lipid sources, amino acids, vitamins/micronutrients, functional additives, and microorganism-based strategies. Controlled feeding trials should incorporate tank-level replication, randomization, experimental-unit definition, water-quality monitoring, and feed/water/biofilm microbiome controls. Molecular profiling should include qPCR, host RNA-seq, absolute microbial quantification, shotgun metagenomics or metatranscriptomics, microbiota analysis, metabolomics, lipidomics, proteomics, and culturomics, supported by RNA-quality assessment, primer validation, reference-gene validation, pathway statistics, and data deposition. Phenotypic integration should include growth, FCR, feed efficiency, histology, immunity, antioxidant status, pathogen load, disease challenge, and environmental stress tolerance. Pathway consistency, microbial function, biological relevance, tissue specificity, phenotype alignment, and species specificity should then be used to generate sustainable, efficient, healthy, and climate-resilient precision aquafeed recommendations.

Computational and digital technologies can extend this framework. AI-assisted biomarker discovery and machine-learning models can integrate multi-omic data with diet composition, environmental conditions, and production phenotypes to identify predictive nutritional signatures. Genome-scale metabolic modeling can translate transcriptomic and metabolomic information into testable predictions of nutrient flux and host–microbial metabolic interactions. Digital phenotyping based on imaging, video, acoustic monitoring, or biosensors can continuously quantify feeding behavior, growth, activity, and stress-related traits, allowing molecular biomarkers to be aligned with real-time phenotypes. These approaches require standardized datasets, transparent feature selection, model interpretation, independent validation, and farm-scale testing before routine application. An integrated framework for future precision aquafeed nutrigenomics is presented in Figure 5.

Microbiome-specific knowledge gaps are particularly important. Most studies profile bacteria using relative-abundance 16S rRNA data, use limited numbers of pooled samples, and do not consistently distinguish digesta from mucosa or account for feed, water, and biofilm communities. Fungal, viral, archaeal, strain-level, and microbial functional responses also remain poorly characterized.

Future studies should therefore combine absolute microbial quantification with shotgun metagenomics or metatranscriptomics, direct metabolite measurements, culture-based validation, longitudinal sampling, and pathogen-load assays. Strain-resolved characterization, cultured isolates, defined microbial communities, and challenge models will be particularly important for distinguishing causal microbial mediators from transient dietary passengers or biomarkers of host condition [27,28,29].

The proposed workflow emphasizes that molecular and microbial biomarkers should be validated across genotypes, life stages, production systems, and environmental conditions before their application to practical aquafeed formulation. Experimental designs should also define the tank-level unit, report randomization and water quality, include feed, water, and biofilm controls, validate qPCR and RNA-seq methods, deposit sequencing data, and specify pathway-analysis procedures.

Future precision aquafeed studies should integrate gene expression with nutrient digestibility, growth, feed utilization, tissue composition, histology, plasma biomarkers, antioxidant activity, immune assays, microbiota, metabolomics, lipidomics, proteomics, disease challenge, and environmental stress tests [10,11,13,37,43]. Species- and stage-specific reference resources are also required because responses observed in rainbow trout, gilthead sea bream, common carp, grouper, or sole cannot be generalized automatically to other cultured species.

Reproducibility should be evaluated at multiple levels. Technical reproducibility concerns concordance across assays or analytical platforms, biological reproducibility concerns agreement among independent tanks, cages, experiments, or cohorts, and external reproducibility concerns persistence across facilities, populations, life stages, production systems, and environmental conditions. Future studies should distinguish these forms of validation explicitly, balance treatments across analytical batches, retain the experimental unit in molecular and statistical models, apply appropriate multiple-testing correction, and provide raw data, metadata, and analysis workflows sufficient for independent reanalysis. Replication of the principal pathway and phenotype associations in an independent experiment would provide stronger evidence than confirmation of selected transcripts using the original samples alone.

Industrial translation requires evaluation beyond biological efficacy. Cost-effectiveness should include ingredient and additive prices, analytical and quality-control costs, manufacturing, storage, transportation, and the feed cost required to produce a unit of marketable biomass. Commercial scalability also depends on reliable raw-material supply, batch consistency, palatability, nutrient stability, shelf life, and compatibility with grinding, mixing, extrusion, pelleting, drying, coating, and storage. For probiotics and other microorganism-based products, viable dose, thermal and mechanical stability, contamination control, and stability throughout feed storage and delivery are additional manufacturing constraints.

Regulatory approval, farmer adoption, and farm-level economic return should be considered early in development. Regulatory evaluation may require documented ingredient identity, composition, safety, contaminants, antimicrobial-resistance risks, manufacturing consistency, and evidence supporting nutritional or functional claims. Adoption will also depend on compatibility with existing feeding practices, ease of use, technical support, product availability, perceived production risk, and demonstrated benefits under commercial conditions. Candidate precision feeds should therefore be validated in multi-site and commercial-scale trials across seasons, stocking densities, production systems, and environmental conditions. Economic assessments should compare feed cost per kilogram of weight gain, feed conversion ratio, survival, disease-related losses, product quality, and net return with those of standard commercial diets. A formulation should be considered commercially promising only when its biological benefits are reproducible and sufficient to offset any additional ingredient, processing, monitoring, or regulatory costs.

Overall, the most promising research directions are causal, cell- and spatially resolved, computationally integrated, standardized, and translational. Replicated time-series studies, harmonized analytical pipelines, interpretable predictive models, independent validation, and farm-scale testing will be essential for converting multi-omic discoveries into species-specific precision aquafeed recommendations that improve nutrient utilization, intestinal health, disease resistance, and environmental resilience.

## 7. Conclusions

Well-supported evidence indicates that alternative-protein and lipid-source interventions can modify host molecular and physiological responses in cultured finfish. Recurrent responses involve intestinal inflammation and epithelial-barrier integrity, nutrient transport, lipid and bile-acid metabolism, long-chain polyunsaturated fatty-acid biosynthesis, amino-acid transport and growth signaling, antioxidant regulation, tissue composition, and growth-related outcomes. However, the direction and magnitude of these responses depend strongly on the species, life stage, basal diet, ingredient processing, dietary dose, sampled tissue, exposure duration, and experimental design; therefore, universal formulation thresholds cannot yet be inferred.

Microorganisms are likely important mediators at the diet–host interface because dietary interventions were repeatedly associated with changes in intestinal microbial communities, host gene expression, intestinal condition, metabolism, and selected disease-related outcomes. Nevertheless, direct causal evidence for microbial mediation in cultured finfish remains limited. Most available studies relied on 16S rRNA relative-abundance profiles, taxonomic correlations, or predicted microbial functions, and did not demonstrate that microbial shifts or microbial metabolites caused the observed host responses. Microbial changes should therefore be interpreted as candidate mechanisms or biomarkers unless temporal precedence, microbial activity, metabolite production, and causal contribution are demonstrated directly.

Accordingly, current evidence supports evaluating aquafeeds using appropriately replicated experiments and concordant molecular, biochemical, histological, microbial, and phenotypic outcomes. Molecular or taxonomic changes alone should not be regarded as proof of nutritional benefit or microbial mediation. Causal microbiome manipulation and advanced multi-omic and computational approaches remain important research priorities rather than established requirements for commercial precision aquafeed formulation.

## Figures and Tables

**Table 2:** Pathway-based synthesis and evidence levels for diet-associated nutrigenomic responses in cultured finfish.

Biological Pathway	Principal Dietary Evidence	Directly Measured Outcomes	Evidence Level	Principal Limitation	References
Intestinal inflammation and epithelial-barrier integrity	Soybean meal, fermented soybean meal, soybean-protein concentrate, tributyrin, phytobiotics, and probiotics	Intestinal transcripts, histology, digestive enzymes, immune and oxidative markers, and challenge outcomes.	L1—Replicated controlled evidence	Evidence is concentrated in soybean-sensitive carnivores; Studies 4–6 may share controls, and transcript changes may not indicate functional improvement.	[17,18,20,21,22,26,39,40,41]
Lipid, cholesterol, LC-PUFA, and bile-acid metabolism	Long-term plant-based diets, graded n-3 HUFAs, parental lipid programming, and taurine-supplemented plant-based diets	*fads2*, *elovl5*, lipid-transport transcripts, tissue lipids, bile acids, hepatic lipid deposition, plasma triacylglycerol, and growth.	L1—Replicated controlled evidence	Species, life stage, lipid source, and tissue varied, and several datasets were targeted or graphically reported rather than multi-omic.	[7,14,15,16,32]
Amino-acid transport and TOR/IGF growth signaling	Alternative proteins and graded or deficiency-model methionine studies; selected phytobiotic interventions	Amino-acid transporter and TOR/IGF-related transcripts; TOR-pathway phosphoproteins; protein retention; growth and feed utilization	L1—Replicated controlled evidence	Evidence is derived from a limited range of species and mainly targeted pathway analyses; several tissue samples were pooled at the tank or cage level.	[19,30,31,39]
Antioxidant and immune regulation	Vitamin E, tributyrin, *Bidens pilosa*, and single- or multi-strain probiotics	*nrf2*/*keap1*-related transcripts; antioxidant-enzyme activities; MDA; IgM; cytokines; innate immune markers; challenge survival	L1—Replicated controlled evidence	Responses were context-dependent, with limited protein or pathway-activity validation.	[26,34,39,40,41]
Diet-associated microbial ecology and host-response covariation	Soybean-derived proteins, fishmeal-free formulations, tributyrin, phytobiotics, and probiotics	16S profiles, diversity, taxon–host correlations, transcriptomics, histology, metabolites, and disease outcomes.	L2—Integrated but limited-replication evidence	Most studies used relative-abundance 16S data, with limited assessment of viability, absolute abundance, environmental sources, or temporal dynamics.	[22,24,25,26,39,40,41]
Microbial metabolite production and functional mediation	Tributyrin, plant-based diets, phytobiotics, and predicted microbial metabolic functions	Selected intestinal metabolites; barrier and immune transcripts; histology; host transcriptomics; 16S-based functional predictions	L3—Molecular or marker-based evidence	Metabolite production was often inferred, with limited evidence of microbial flux, host exposure, or causal mediation	[22,26,39]
Disease resistance and pathogen or parasite outcomes	Fishmeal-free functional diets and probiotic interventions	*Nocardia* challenge survival; parasite susceptibility; immune transcripts; selected microbial-community changes	L2—Integrated but limited-replication evidence	Few pathogen or parasite models were included, with strain- and pathogen-specific effects and non-independent pre-challenge sampling in Study 21.	[24,40,41]
Epigenetic and nutritional programming	Broodstock lipid source and selection for high or low *fads2* expression	Parental *fads2* expression; offspring promoter methylation; hepatic lipid-related expression; fatty-acid phenotype; growth	L2—Integrated but limited-replication evidence	Evidence derives mainly from one complex broodstock–offspring study with small methylation subgroups and limited cross-species replication.	[15]
Environmental stress resilience	Graded taurine supplementation followed by acute and lethal thermal exposure	Cortisol and other plasma indices; antioxidant and immune responses; heat-shock transcripts; survival	L2—Integrated but limited-replication evidence	One feeding study showed modest dietary effects, unchanged heat-shock transcripts, and no improvement in lethal-stress survival.	[33]

Abbreviations: 16S, bacterial 16S ribosomal RNA gene sequencing; IGF, insulin-like growth factor; IgM, immunoglobulin M; LC-PUFA, long-chain polyunsaturated fatty acid; MDA, malondialdehyde; n-3 HUFA, omega-3 highly unsaturated fatty acid; TOR, target of rapamycin. Footnote: L1—Replicated controlled evidence that indicates support from several independent controlled feeding studies, with concordance between molecular responses and one or more downstream functional or phenotypic outcomes. L2—Integrated but limited-replication evidence that indicates support principally from one integrated controlled study or from a small evidence base with limited independence but multiple evidence layers. L3—Molecular or marker-based evidence that indicates support mainly from gene-expression, transcriptomic, 16S rRNA, taxonomic-association, or predicted-function data without direct functional or causal validation. These levels are descriptive synthesis categories and do not constitute formal risk-of-bias or study-quality grades. Studies 4–6 were not counted as fully independent confirmations because of potentially overlapping or reused control data.

**Table 3:** Methodological and reporting priorities for future finfish nutrigenomics and microbiome studies.

Domain	Recommended Practice	Why It Matters	Minimum Reporting Elements
Diet formulation and analytical verification	Report diet formulation, nutrient composition, ingredient batches, inclusion levels, and feed processing.	Molecular responses cannot be attributed confidently when dietary exposures are poorly defined.	Ingredient sources; formulation; proximate composition; relevant amino-acid and fatty-acid profiles; additive stability.
Experimental unit and statistical power	Use the tank or cage as the experimental unit, distinguish subsamples from true replicates, and justify sample size.	Pseudoreplication inflates precision, whereas too few independent units reduce statistical power.	Report treatment units, fish numbers, sampling, pooling, replicates, power rationale, and statistical model.
Randomization and tank positioning	Randomize treatments and control tank-position effects by blocking or rotation.	Light, flow, temperature, and location gradients can confound dietary effects.	Allocation method; block structure; tank map or rotation; relevant environmental covariates.
Blinding and sample handling	Blind assessments and standardize sampling and processing conditions.	Pre-analytical variation can equal or exceed true diet-induced molecular effects.	Sampling order; fasting period; circadian time; blinding; preservation method; storage duration.
qPCR validation	Follow MIQE guidelines, validate primers and reference genes, and specify the validation type.	Unstable reference genes or poor assay performance can distort apparent fold changes.	Report primer performance, RNA quality, reference-gene stability, normalization, and replicates.
RNA-seq, batch control, and pathway analysis	Use sufficient biological replicates, balanced batches, multiple-testing correction, and validation.	Insufficient replication, batch effects, and thresholds can mislead; read depth cannot replace biological replication.	Report library design, RNA quality, sequencing metrics, statistical thresholds, pathway database, and accession number.
Reproducibility and data transparency	Deposit data, metadata, code, and protocols, and specify the validation type.	Transparent reporting enables independent reanalysis and cross-study evaluation.	Report data repositories, raw data, metadata, software, code, parameters, and validation status.
Multi-omic and phenotype integration	Integrate molecular data with matched biochemical, histological, microbial, and production outcomes.	Agreement across independent evidence strengthens biological interpretation.	Report matched samples, batch control, cross-omic integration, concordance, and external validation.

Abbreviations: MIQE, Minimum Information for Publication of Quantitative Real-Time PCR Experiments; qPCR, quantitative polymerase chain reaction; RNA-seq, RNA sequencing.

**Table 4:** Microorganism-centered mechanisms connecting aquafeed composition with host nutrigenomic responses in cultured finfish.

Microbial Dimension	Representative Organisms or Functions	Dietary Mechanism	Host Molecular/Functional Readouts	Interpretation Priorities
Diet-driven community succession	Lactic-acid bacteria, *Cetobacterium*, *Microbacterium*, *Mycoplasma*, and other context-dependent taxa	Changes in protein, fiber, lipid, bile acids, antinutritional factors, and feed-associated inocula	Cytokines, tight-junction genes, nutrient transporters, histology, growth	Do not equate diversity or single taxa with health; distinguish digesta, mucosa, feed, and water sources.
Lactic-acid-bacterial probiotics	*L. rhamnosus*; *Limosilactobacillus reuteri*	Organic acids, pathogen antagonism, adhesion competition, immune modulation	*il1beta*, *tnfalpha*, *il8*, *ifng*, barrier markers, pathogen burden, survival	Effects are strain and dose specific; verify viability, colonization/activity, and challenge protection [41].
Spore-forming or enzymatic probiotics	*Bacillus subtilis*; strain-specific *Bacillus* and *Exiguobacterium* preparations	Digestive enzymes, antimicrobial products, nutrient release, community restructuring	Digestive enzymes, IGF/myogenic genes, and antioxidant and immune markers	Use strain-level identification and screen for toxin, virulence, and resistance genes, especially in the *B. cereus* group [40].
Microbial metabolites and postbiotics	Butyrate/tributyrin, acetate, propionate, lactate, bile-acid transformations	Fermentation and microbial bioconversion of dietary substrates	*zo-1*, *occludin*, mucins, cytokines, epithelial energy, and hepatic metabolism	Measure metabolites directly and avoid inferring production solely from 16S profiles [26].
Fermented ingredients and microbial biomass	Fermentation organisms, yeasts, fungi, bacteria, microalgae	Reduce antinutritional factors and release bioactive metabolites and cell-wall signals.	Barrier integrity, immunity, digestibility, nutrient sensing, growth	Report organism, viability, metabolites, batch consistency, and nutritional or bioactive role.
Pathobionts and pathogens	*N. seriolae*; context-dependent *Mycoplasma*; *Vibrio*/*Aeromonas* at the strain level	Barrier disruption, ecological release, stress-enhanced virulence, or competitive exclusion	Virulence, pathogen load, tissue invasion, inflammation, morbidity, and survival.	Taxonomic presence does not indicate disease; use strain-level and challenge evidence.
Environmental microbiome	Feed, water, biofilm, sediment, and system-associated microorganisms	Colonization pressure and continual exchange with host surfaces	Microbial load, community assembly, water quality, host mucosal responses	Treat tanks as ecological units; include environmental controls and account for shared-water transmission.

Abbreviations: IGF, insulin-like growth factor; 16S rRNA, 16S ribosomal RNA.

**Table 5:** Summary of major dietary interventions, molecular pathways, microbiome responses, and practical implications in cultured finfish nutrigenomics.

Practical Implications for Aquaculture	Principal Microbiome Responses	Key Molecular Pathways and Markers	Major Dietary Intervention	References
Define species-specific inclusion and processing limits based on growth, gut health, and microbiota.	High inclusion may impair microbiota and barrier integrity; processing, species, and dose influence responses.	Assess immune signaling, epithelial-barrier markers, nutrient and cholesterol metabolism, and miRNA–mRNA regulation.	Alternative proteins: soybean-based, insect, microbial, and algal proteins.	[7,8,9,17,18,19,20,21,22]
Sustainable lipid replacement: preserve EPA/DHA, fillet quality, liver health, bile metabolism, and growth.	Microbiome evidence is limited; lipid, taurine, and bile-acid changes may influence microbial selection and metabolism.	Lipid catabolism: *ppara*, *cpt1*; lipogenesis: *srebp1*, *fas*; LC-PUFA synthesis: *fads2*, *elovl5*; bile-acid metabolism: *cyp7a1*, *abcb11*.	Lipid interventions: fish oil replacement, vegetable/algal oils, n-3 HUFA, and taurine-supplemented plant diets.	[3,14,15,16,25,32]
Validate strain identity, safety, stability, dose, and feed persistence; efficacy alone does not ensure safety.	Microbial shifts are strain and context specific, while metabolite production and pathogen protection require direct validation.	Barrier and mucin regulation; cytokine, antioxidant, and IGF-myogenic signaling; epithelial metabolism; host–microbiota interactions.	Functional additives: probiotics, microbiota-targeted substrates, postbiotics, phytobiotics, and fermented ingredients.	[23,24,25,26,39,40,41]
Use species-specific dose–response trials to assess growth, amino-acid utilization, nutrient sensing, and protein retention.	Direct microbiome evidence is limited; taurine-mediated bile-acid changes may influence antimicrobial pressure and host–microbiota signaling.	Amino-acid transport via ASCT2; TOR/S6K, 4E-BP1, and IGF-I signaling; methyl metabolism; antioxidant capacity; and bile-acid and lipid utilization.	Amino-acid supplementation, particularly methionine and taurine.	[30,31,32,33]
Determine optimal inclusion using molecular, enzymatic, oxidative, immune, growth, survival, and toxicity indicators.	Microbiome responses may be indirectly mediated by redox balance, barrier integrity, and mucosal immunity.	Nrf2/Keap1 signaling; antioxidant and detoxification genes such as *sod*, *cat*, *gpx*, *gst*, and *gr*; immune and membrane-protection pathways	Vitamins and minerals, particularly vitamin E and other antioxidant-related micronutrients.	[4,6,12,34]
Validate dietary resilience across life stages and realistic stressors; molecular compensation alone does not prove protection.	Environmental stress may destabilize microbiota and increase pathobiont risk, but diet–microbiome links to stress resilience remain limited.	Stress markers (*hsp60*, *hsp70*, *hsp90*, and *wap65*); antioxidant and endocrine responses; *fads2* expression and methylation; metabolic programming.	Nutritional programming and stress-modulating diets.	[10,13,15,33]

Abbreviations: 4E-BP, eukaryotic translation initiation factor 4E-binding protein; ASCT2, alanine–serine–cysteine transporter 2; DHA, docosahexaenoic acid; EPA, eicosapentaenoic acid; GPx, glutathione peroxidase; HUFA, highly unsaturated fatty acid; IGF-I, insulin-like growth factor I; LC-PUFA, long-chain polyunsaturated fatty acid; miRNA, microRNA; mRNA, messenger RNA; n-3, omega-3; Nrf2/Keap1, nuclear factor erythroid 2-related factor 2/Kelch-like ECH-associated protein 1; TOR/S6K, target of rapamycin/ribosomal protein S6 kinase; *abcb11*, ATP-binding cassette subfamily B member 11; *cat*, catalase; *cpt1*, carnitine palmitoyltransferase 1; *cyp7a1*, cholesterol 7α-hydroxylase; *elovl5*, elongation of very-long-chain fatty-acid protein 5; *fads2*, fatty-acid desaturase 2; *fas*, fatty-acid synthase; *gpx*, glutathione peroxidase; *gr*, glutathione reductase; *gst*, glutathione S-transferase; *hsp60*, *hsp70*, and *hsp90*, heat-shock proteins 60, 70, and 90; *ppara*, peroxisome proliferator-activated receptor alpha; *sod*, superoxide dismutase; *srebp1*, sterol regulatory element-binding protein 1; *wap65*, warm-temperature-acclimation-related 65 kDa protein.

## Data Availability

No new experimental data were generated in this review. All data analyzed were derived from previously published studies cited in the article. The study-level data extracted for the structured narrative synthesis are provided in the article and Supplementary Tables S1 and S2, including public data accession information where available.

## Supplementary Materials

The following supporting information can be downloaded at: https://www.mdpi.com/article/10.3390/microorganisms14081786/s1, Table S1: Detailed within-study quantitative outcomes from representative feeding-study reports included in the review; Table S2: Detailed molecular platforms, library construction, sequencing quality, statistical criteria, and data availability.
