# Supplementary material for: Precision Nutrigenomics in Cultured Finfish: Dietary Regulation of Gene Expression, Microbial Ecology, Metabolism, and Immunity
Source: Microorganisms. 2026 Aug 13;14(8):1786. doi: 10.3390/microorganisms14081786 (PMC13515195; doi:10.3390/microorganisms14081786)
Supplement: Supplementary file 1 [file microorganisms-14-01786-s001.zip › Supplementary_Table_S1.pdf]

Supplementary Table S1. Detailed within-study quantitative outcomes from representative feeding-study reports included in the review

Study-design characteristics, biological sampling, experimental units, analytical methods, and principal limitations are summarized in Table 1. This supplementary table provides the detailed dietary comparisons and quantitative outcomes used in the narrative synthesis.

Species	Detailed dietary comparison	Quantitative production outcomes	Quantitative functional, physiological, or histological outcomes	Quantitative molecular or microbiome outcomes	References
Japanese seabass (*Lateolabrax japonicus*)	Fishmeal diet vs 50% or 75% fishmeal replacement with soybean meal (FM, SM50, SM75)	SM50 maintained FBW and WG statistically, but FE fell from 0.92 to 0.86. SM75 reduced FBW from 59.10 to 38.83 g, WG from 1078% to 799%, SGR from 4.40 to 3.74%/d, and survival from 100% to 78.8%.	SM75 reduced foregut protease from 82.94 to 22.39 U/mg protein and foregut villus height from 443.22 to 287.83 µm; inflammatory genes increased and *IL-4* decreased.	Targeted qPCR of inflammatory and nutrient-transporter genes; exact transcript fold changes were graphically reported rather than tabulated.	[17]
Yellow perch (*Perca flavescens*)	Fishmeal diet vs diet replacing 75% of fishmeal protein with soybean meal	SBM reduced final weight from 5.11 to 4.11 g and absolute weight gain from 4.98 to 3.98 g; survival was 91% vs 84% (not significant).	Whole-body lipid and cholesterol did not differ between diets.	1,025 transcript isoforms were differentially expressed ($p < 0.05$); 53 isoforms representing 33 genes changed by at least 3-fold, mainly in cholesterol biosynthesis.	[7]
Hybrid grouper (*E. fuscoguttatus* ♀ × *E. lanceolatus* ♂)	0%, 10%, 30%, or 50% fishmeal protein replacement with soybean meal	SBM50 reduced WGR from 462.46% to 383.76% and increased FCR from 0.96 to 1.14; survival remained statistically unchanged (100% vs 98.89%).	SBM50 significantly reduced distal-intestinal plica height, muscular-layer thickness, and goblet-cell number.	6,390 DEGs (3,330 up; 3,060 down) and 92 differentially expressed miRNAs were identified in SBM50 vs FM.	[18]
Pearl gentian grouper (*E. fuscoguttatus* ♀ × *E. lanceolatus* ♂)	Fishmeal control vs 20% or 40% fishmeal protein replacement with fermented soybean meal	FSBM40 reduced WGR from 485.14% to 427.22% and increased FCR from 0.84 to 0.95; survival remained approximately 99%.	FSBM40 increased the mucosal-fold enteritis score from 1.27 to 4.17, trypsin from 597.33 to 1194.50 U/mg, and MDA from 2.49 to 4.15 nmol/mg, while IgM fell from 94.33 to 50.84 µg/mg.	FSBM40 increased *TNF-α* expression from 1.01 to 4.09-fold and reduced *TGF-β1* from 1.00 to 0.13-fold; 3,462 DEGs were detected.	[20]
Pearl gentian grouper (*E. fuscoguttatus* ♀ × *E. lanceolatus* ♂)	Fishmeal control vs 20% or 40% fishmeal protein replacement with soybean meal	SBM40 reduced FBW from 73.82 to 66.08 g and WGR from 485.14% to 426.50%, while FCR increased from 0.84 to 0.95.	SBM40 increased the mucosal-fold enteritis score from 1.27 to 4.87, MDA from 2.49 to 4.25, and observed bacterial species from 92.33 to 909.33; Shannon diversity rose from 2.30 to 6.27.	2,305 DEGs were detected in SBM40 vs FM; metabolomics identified hundreds of differential intestinal-content metabolites and 56 metabolites exchanged between intestinal tissue and contents.	[22]
Pearl gentian grouper (*E. fuscoguttatus* ♀ × *E. lanceolatus* ♂)	Fishmeal control vs 20% or 40% fishmeal protein replacement with soybean protein concentrate	SPC40 reduced FBW from 73.82 to 66.73 g and WGR from 485.14% to 431.73%, while FCR increased from 0.84 to 0.94.	SPC40 reduced plica height/width from 7.80 to 4.82 and microvillus length from 24.10 to 14.98 µm; apoptotic-cell number rose from 12.75 to 51.75 and IgM fell from 94.33 to 37.77 µg/mg.	SPC40 reduced *TGF-β1* expression from 1.00 to 0.18-fold; 4,076 DEGs were identified (2,328 upregulated and 1,748 downregulated). †	[21]

Species	Detailed dietary comparison	Quantitative production outcomes	Quantitative functional, physiological, or histological outcomes	Quantitative molecular or microbiome outcomes	References
Grass carp (*Ctenopharyngodon idellus*)	Complete replacement of soybean meal with CAP, *Tenebrio molitor* meal, cottonseed protein concentrate, or chlorella powder	In small fish, CHP produced numerically higher WGR (132.13% vs 118.38%) and lower FCR (1.50 vs 1.64) than SBM, but these differences were not significant. TMM produced numerically lower WGR (85.95%) and higher FCR (2.40) than SBM, also without significant differences from SBM. In large fish, no alternative produced significantly greater growth than SBM.	CHP improved muscle protein, texture, water-holding traits, and amino-acid composition relative to several alternatives.	In small fish, CHP increased muscle *TOR* expression from 1.30 to 3.06 and *S6K* from 1.35 to 1.92; growth-regulatory gene responses were size dependent.	[19]
Rainbow trout (*Oncorhynchus mykiss*)	Marine diet, commercial-like partially plant-based diet, or fully plant-based diet from first feeding	At 13 months, fully plant-fed fish weighed 234 g vs 330–337 g in the other diets. FE was numerically lower (1.04 vs 1.19–1.22) but was not statistically different ($p = 0.11$). Juvenile survival at 7 months was 65% vs approximately 95%.	Juvenile whole-body lipid increased to 13.2% vs 9.4–9.9%; DHA fell to 2.2% vs 14.5% in the marine group. Plasma cholesterol was about twofold lower with plant-based diets.	Intestinal protein-catabolism/carbohydrate genes were downregulated in juveniles; hepatic lipid/cholesterol genes were upregulated at both stages.	[14]
Gilthead sea bream (*Sparus aurata*)	Broodstock selected for high/low blood *fads2* expression and fed fish oil or high-vegetable-oil diets; offspring challenged with low-FM/FO diet	Offspring from high-*fads2* parents had significantly higher SGR than low-*fads2* offspring; exact SGR means were presented graphically. HSI ranged from 1.2% to 1.6%.	Offspring from low-*fads2* parents accumulated more selected fatty-acid substrates/products and showed altered hepatic lipid metabolism.	Female parental *fads2* expression was 5.77-fold vs 0.17-fold, and male expression was 3.05-fold vs 0.26-fold in groups subsequently assigned to the fish oil diet; offspring promoter methylation reflected parental expression and its interaction with broodstock diet.	[15]
Golden pompano (*Trachinotus ovatus*)	Five diets containing 0.64%, 1.00%, 1.24%, 1.73%, or 2.10% n-3 HUFA	Growth and feed-utilization outcomes were not reported in the article.	Muscle TAG represented 72.47–84.53% of total lipid, PC 3.41–6.70%, and PE 1.98–2.71%; EPA, DPA, DHA, and total n-3 HUFA increased across TAG, PC, and PE with dietary HUFA.	126,792 unigenes were assembled; 47.78% matched Swiss-Prot, and 232 DEGs (119 up; 113 down) were identified between D2 and D5 in later analysis.	[16]
Common carp (*Cyprinus carpio*)	Fishmeal control and all-plant diets supplemented with 0.5, 1.0, 2.0, or 4.0 g/kg tributyrin	Compared with the low-TB all-plant diet (D2), D5 increased WG from 152.4% to 179.73%, SGR from 1.65 to 1.84%/d, and reduced FCR from 1.24 to 1.16.	D5 increased intestinal trypsin from 35.43 to 50.25, lipase from 265.19 to 327.82, and amylase from 161.19 to 242.61 U/mg protein; villus development improved.	High TB upregulated antioxidant, anti-inflammatory, tight-junction, and villification genes and altered microbiota, including recovery of Proteobacteria and Actinobacteria.	[26]

Species	Detailed dietary comparison	Quantitative production outcomes	Quantitative functional, physiological, or histological outcomes	Quantitative molecular or microbiome outcomes	References
Gilthead sea bream (*Sparus aurata*)	Conventional control vs fishmeal-free circular formulation with alternative proteins, oils, and health additive	Final weight was 43.92 vs 43.02 g (NS); SGR decreased from 0.0233 to 0.0227 day^{-1}, and whole-tank FCR was 0.93 vs 0.97.	Intestinal weight index increased from 4.15% to 4.65%; at 78 dpe, parasite prevalence and intensity did not differ, and disease outcomes were similar.	17, 2, and 4 genes differed in liver, head kidney, and posterior intestine; observed OTUs increased from 207.56 to 257.89 and ACE from 306.76 to 375.06.	[24]
Gilthead sea bream (*Sparus aurata*)	Reference vs growth-selected fish fed control or FUTURE low-marine-resource diets across a production cycle	Estimated feed intake was 0.68–0.85 g/100 g BW/day and did not differ among treatments; detailed growth outcomes were reported in a companion study.	1,892 OTUs were identified; microbiota succession was driven strongly by developmental stage and secondarily by genotype and diet.	1,429 transcripts changed across comparisons; 1,018 significant associations linked 96 OTUs with 476 DE transcripts.	[25]
Cobia (*Rachycentron canadum*)	Seven low-fishmeal diets containing 0.72–1.86% methionine	At 1.24% Met, WGR was 544.32% vs 474.74% at 0.72%, SGR 3.33 vs 3.12%/d, and FCR 1.68 vs 1.97.	The estimated optimal Met requirement was 1.24% of diet (2.71% dietary protein).	At 1.24% Met, muscle *IGF-I* expression was 3.09 vs 1.00 and *TOR* 1.54 vs 1.00; TOR Ser2448 phosphorylation was highest.	[30]
Largemouth bass (*Micropterus salmoides*)	Adequate methionine (1.21%) vs methionine-deficient diet (0.93%)	Adequate Met increased FBW from 21.9 to 26.9 g, WGR from 438% to 557%, SGR from 3.01 to 3.36%/d, FER from 0.88 to 1.12, and PER from 1.83 to 2.28.	Protein retention increased from 33.0% to 43.3%; plasma total essential amino acids increased from 152 to 266 μg/mL.	Adequate Met increased TOR-pathway transcription and phosphorylation of AKT, S6, and 4E-BP1 while suppressing the amino-acid-response pathway.	[31]
Senegalese sole (*Solea senegalensis*)	Fishmeal diet, plant-protein diet, and plant diet supplemented with 0.5% or 1.5% taurine	PP1.5 restored WG to 98.9%, compared with 77.2% in PP0 and 102.6% in FM; FCR did not differ significantly.	PP0 reduced tissue taurine and bile-acid concentration and increased hepatic fat and plasma triglycerides relative to FM. Taurine supplementation partially mitigated these effects: PP1.5 was not different from FM for hepatic fat or plasma triglycerides. Whole-body fat was lower in all plant-diet groups and was not significantly affected by taurine supplementation (19.9% DM in FM; 13.7% in PP1.5).	PP0 downregulated *cyp7a1* and *abcb11* relative to FM. PP0.5 increased both transcripts relative to PP0 but remained different from FM; PP1.5 did not differ significantly from PP0 or PP0.5. Thus, full restoration to the FM expression level was not demonstrated.	[32]
Silver sillago (*Sillago sihama*)	Six diets containing 4, 14, 22, 47, 95, or 198 mg/kg vitamin E	At 95 mg/kg, WG was 619.60% vs 530.18% at 4 mg/kg, SGR 3.52 vs 3.33%/d, and FCR 0.94 vs 1.04.	At 95 mg/kg, hepatic IgM increased from 48.40 to 83.57 μg/mg protein and MDA decreased from 13.29 to 8.19 nmol/mg protein.	Vitamin E upregulated *Nrf2*/*Keap1*-related antioxidant genes; regression estimated 100.37 mg/kg for WG, 116.37 mg/kg for IgM, and 114.90 mg/kg for MDA.	[34]
Olive flounder (*Paralichthys olivaceus*)	Seven diets containing 0.13–2.87% taurine, followed by acute (29 °C) and lethal (31 °C) thermal tests	Growth and survival did not differ significantly; whole-body lipid declined from 4.09% at 0.13% taurine to 3.06% at 2.87%.	Under acute heat stress, cortisol was 46.55 ng/mL at 0.13% taurine vs 2.04 ng/mL at 2.54%; lethal-stress survival did not differ among diets.	Brain and liver *hsp60*, *hsp70*, *hsp90*, and *wap65* expression did not differ among taurine treatments.	[33]

Species	Detailed dietary comparison	Quantitative production outcomes	Quantitative functional, physiological, or histological outcomes	Quantitative molecular or microbiome outcomes	References
Tilapia (*Oreochromis* spp.)	Control diet vs 0.5% or 1.0% *Bidens pilosa*	At 1% BP, FBW increased from 39.56 to 53.69 g, PWG from 435.77% to 632.40%, SGR from 3.00 to 3.55%/d, FCR fell from 1.72 to 1.43, and survival rose from 82.22% to 100%.	At 1% BP, serum triglycerides decreased from 173.10 to 118.93 mg/dL and cholesterol from 80.21 to 58.78 mg/dL.	Liver DEGs numbered 1,228 (0.5%) and 1,431 (1%); muscle DEGs numbered 1,138 and 845, respectively; growth-related genes and microbiome functions were integrated.	[39]
Hybrid grouper (*E. fuscoguttatus* ♀ × *E. lanceolatus* ♂)	Control; *B. cereus*; *E. acetylicum*; 1:1 mixed strains; *L. acidophilus* positive reference	Probiotic supplementation significantly improved WG, WGR, and SGR relative to the control, and the mixed-strain group showed the highest growth values. Exact growth and survival means were presented graphically; survival did not differ significantly among groups.	Mixed probiotics produced the highest amylase, lipase, and pepsin activities and enhanced antioxidant and immune indices.	Mixed probiotics upregulated growth- and immune-related genes, did not significantly alter microbiota richness/diversity, enriched *Cetobacterium* and *Microbacterium*, and reduced *Mycoplasma*.	[40]
Grey mullet (*Mugil cephalus*)	Control vs *L. rhamnosus* FS3051, *L. reuteri* FS3052, or *B. subtilis* natto NTU-18 (10^9 CFU/g diet)	*B. subtilis* produced the numerically highest WGR (58.63% vs 25.99%), FE (69.80% vs 30.94%), and SGR (1.63 vs 0.82%/d). All probiotic groups were significantly higher than the control and did not differ significantly from one another.	After *Nocardia seriolae* challenge, survival was 70% with *L. rhamnosus* vs 35% in control; *L. reuteri* and *B. subtilis* each achieved 60% (not significant vs control).	*L. rhamnosus* induced *IL-8*, *IL-1β*, *TNF-α*, *IFN-γ*, and *MHCI* and shifted beta diversity; *Lactobacillus* enrichment correlated positively with growth and immunity.	[41]

Footnote: Values are descriptive within-study comparisons and were not statistically pooled. Statistical significance follows the original article, and a numerical difference is described as significant only when the original comparison supports that conclusion. Exact numerical values were not estimated from figures. NR indicates not reported; GR, graphically reported but not numerically extracted; NC, not directly comparable; and NS, not statistically significant. Study-design details and principal methodological limitations are reported in Table 1; the potential non-independence of Studies 4–6 is addressed there. †The original article reports an inconsistent number of downregulated DEGs between the Results text (1,478) and Table 10 (1,748); the value from Table 10 is presented here because it is consistent with the reported total of 4,076 DEGs.

Abbreviations: 4E-BP1, eukaryotic translation initiation factor 4E-binding protein 1; ACE, abundance-based coverage estimator; AKT, protein kinase B; BP, *Bidens pilosa*; BW, body weight; CAP, *Clostridium autoethanogenum* protein; CFU, colony-forming unit; CHP, chlorella powder; DE, differentially expressed; DEG(s), differentially expressed gene(s); DHA, docosahexaenoic acid; DM, dry matter; DPA, docosapentaenoic acid; EPA, eicosapentaenoic acid; FBW, final body weight; FCR, feed conversion ratio; FE, feed efficiency; FER, feed efficiency ratio; FM, fishmeal; FO, fish oil; FSBM, fermented soybean meal; HSI, hepatosomatic index; HUFA, highly unsaturated fatty acid(s); IBW, initial body weight; IFN-γ, interferon gamma; IGF-I, insulin-like growth factor I; IL, interleukin; IgM, immunoglobulin M; Keap1, Kelch-like ECH-associated protein 1; MDA, malondialdehyde; Met, methionine; MHCI, major histocompatibility complex class I; miRNA(s), microRNA(s); NC, not directly comparable; NR, not reported; Nrf2, nuclear factor erythroid 2-related factor 2; NS, not statistically significant; OTU(s), operational taxonomic unit(s); PC, phosphatidylcholine; PE, phosphatidylethanolamine; PER, protein efficiency ratio; PWG, percentage weight gain; qPCR, quantitative polymerase chain reaction; RNA-seq, RNA sequencing; S6, ribosomal protein S6; S6K, ribosomal protein S6 kinase; SBM, soybean meal; SGR, specific growth rate; SPC, soybean protein concentrate; TAG, triacylglycerol; TB, tributyrin; TGF-β1, transforming growth factor beta 1; TMM, *Tenebrio molitor* meal; TNF-α, tumor necrosis factor alpha; TOR, target of rapamycin; WG, weight gain; WGR, weight gain rate.

Treatment codes: D2 and D5, all-plant diets containing 0.5 and 4.0 g/kg TB, respectively; FSBM40, 40% FM-protein replacement with FSBM; PP0 and PP1.5, plant-protein diets containing 0 and 1.5% taurine, respectively; SBM40 and SBM50, 40 and 50% FM-protein replacement with SBM, respectively; SM50 and SM75, 50 and 75% FM replacement with SBM, respectively; SPC40, 40% FM-protein replacement with SPC.
