# Supplementary material for: Precision Nutrigenomics in Cultured Finfish: Dietary Regulation of Gene Expression, Microbial Ecology, Metabolism, and Immunity
Source: Microorganisms. 2026 Aug 13;14(8):1786. doi: 10.3390/microorganisms14081786 (PMC13515195; doi:10.3390/microorganisms14081786)
Supplement: Supplementary file 1 [file microorganisms-14-01786-s001.zip › Supplementary_Table_S2.pdf]

Supplementary Table S2. Detailed molecular platforms, library construction, sequencing quality, statistical criteria, and data availability

Biological sample numbers, experimental units, principal analytical methods, and study-level limitations are summarized in Table 1; quantitative biological outcomes are reported in Table S1, and pathway-level evidence is summarized in Table 2. This table therefore reports additional molecular-method and data-availability details.

Species	Tissue and molecular endpoint	Platform and analytical workflow	Library construction or pooling	Sequencing scale, quality, and mapping	Molecular or statistical criterion	Public data accession or reporting status	References
Japanese seabass (*Lateolabrax japonicus*)	Foregut and midgut; *PepT1*, *LAT1*, *SLC1A5*, *TNF-α*, *IL-1β*, *IL-2*, *IL-8*, and *IL-4* expression.	SYBR-based RT-qPCR; *β-actin* normalization; 2−ΔΔCt.	No library construction or pooling reported; each sample was analyzed in technical triplicate.	Scale/depth: NA—targeted qPCR study. QC/mapping: RNA concentration/purity by NanoDrop; integrity checked on 1.5% agarose gel; melting-curve specificity.	One-way ANOVA followed by Duncan's multiple-range test; $p < 0.05$.	No sequencing accession applicable.	[17]
Yellow perch (*Perca flavescens*)	Mid-intestine transcriptome; selected differentially expressed genes validated by qPCR.	Illumina HiSeq 3000/4000 RNA-seq, 150-bp paired-end; de novo Trinity assembly; edgeR.	Twelve individual RNA-seq libraries (6/diet); the same RNA was used for qPCR validation.	Scale/depth: Approximately 68 million passing-filter reads per sample. QC/mapping: RIN ≥ 8.7; reads with Phred score < 30 removed; transcripts with mean FPKM < 2 excluded.	edgeR exact test after count normalization; $p < 0.05$; the emphasized biological set additionally required ≥ 3-fold change.	No accession stated in the reviewed main text; processed results and supporting files were supplied with the article.	[7]
Hybrid grouper (*E. fuscoguttatus* ♀ × *E. lanceolatus* ♂)	Distal intestine; mRNA transcriptome and small-RNA/miRNA profiles in FM and SBM50.	BGISEQ-500 mRNA sequencing and small-RNA sequencing; DESeq; integrated miRNA–mRNA analysis.	Three pooled mRNA and three pooled small-RNA libraries/diet; each pool represented one tank.	Scale/depth: mRNA: 72.38–76.50 million clean reads/library. Small RNA: 77,115,163 clean reads for FM and 81,360,125 for SBM50 across the three libraries/group. QC/mapping: mRNA Q30 87.18–88.19%; total mapping 75.76–79.02%; unique mapping 30.94–33.72%. Small-RNA mapping 91.73–94.93%; Q20 approximately 99.0–99.5%.	DE miRNAs were identified using DESeq with $\lvert log2FC \rvert \geq 1$ and Bonferroni-corrected $p \leq 0.01$.	Transcriptome SUB7020170; small-RNA SUB7175134.	[18]
Pearl gentian grouper (*E. fuscoguttatus* ♀ × *E. lanceolatus* ♂)	Distal intestine; full-length transcriptome and targeted qPCR of inflammatory and *TLR-MyD88-NF-κB* pathway genes.	"3+2" strategy: PacBio Sequel full-length sequencing plus Illumina HiSeq 4000 short-read correction; RT-qPCR.	Four tank-level replicates/treatment; exact PacBio/Illumina library pooling was not fully reported.	Scale/depth: NR in the main article; detailed sequencing output was referred to related methodological work. QC/mapping: DE analysis based on corrected full-length transcripts; exact read quality and mapping rates were not reported in the main article.	DE threshold $\lvert log2FC \rvert > 1$ and $p < 0.05$; one-way ANOVA for measured outcomes at $p < 0.05$.	PacBio PRJNA664623; Illumina PRJNA664416.	[20]

Species	Tissue and molecular endpoint	Platform and analytical workflow	Library construction or pooling	Sequencing scale, quality, and mapping	Molecular or statistical criterion	Public data accession or reporting status	References
Pearl gentian grouper (*E. fuscoguttatus* ♀ × *E. lanceolatus* ♂)	Distal intestine and intestinal contents; host transcriptome, 16S rRNA microbiota, metabolomics, qPCR, and Western blot.	PacBio Sequel + Illumina correction; 16S rRNA high-throughput sequencing; LC-MS metabolomics; qPCR; Western blot.	Transcriptomics used 4 replicates/treatment; 16S used 9 samples total; metabolomics used 12 pooled samples/treatment (3 fish/pool).	Scale/depth: 16S: 735,636 raw reads total; group means approximately 79,340–83,851 reads/sample. Host-transcriptome depth NR in this article. QC/mapping: 16S OTUs clustered at 97% identity; Good's coverage > 99%. Host DE analysis used corrected full-length transcripts.	Host DEGs: $\lvert \log2FC \rvert > 1$ and $p < 0.05$. Differential metabolites selected using $p < 0.05$ with VIP-based multivariate criteria.	16S PRJNA666309; PacBio PRJNA664623; Illumina PRJNA664416.	[22]
Pearl gentian grouper (*E. fuscoguttatus* ♀ × *E. lanceolatus* ♂)	Hindgut; full-length transcriptome, 16S microbiota, qPCR, and Western blot.	PacBio Sequel + Illumina HiSeq 4000 correction; 16S rRNA sequencing; RT-qPCR; Western blot.	Four tank-level replicates/treatment; exact host-transcriptome and 16S pooling was not fully reported.	Scale/depth: NR in the main article for both host transcriptome and 16S datasets. QC/mapping: RNA purity/integrity and sequence-processing procedures were described, but numerical Q-scores and mapping rates were not provided in the main article.	DE threshold $\lvert \log2FC \rvert > 1$ and $p < 0.05$; other endpoints compared at $p < 0.05$.	16S PRJNA666309; PacBio PRJNA664623; the article prints the Illumina accession as PRJNA66441, which appears incomplete.	[21]
Grass carp (*Ctenopharyngodon idellus*)	Muscle; *TOR*, *4E-BP1*, *S6K*, *MyoD*, *MyoG*, *Myf5*, *MRF4*, *MSTN-1*, and related growth genes.	ABI 7500 real-time qPCR; *β-actin* normalization; 2−ΔΔCt.	Targeted qPCR; no sequencing-library construction or pooling.	Scale/depth: NA—targeted qPCR study. QC/mapping: Primer standard curves were generated using six cDNA concentrations in triplicate; amplification efficiencies were approximately 100%.	Two-way ANOVA for fish size × protein source, followed by Duncan's multiple-range test; $p < 0.05$.	No sequencing accession applicable.	[19]
Rainbow trout (*Oncorhynchus mykiss*)	Juvenile intestine and liver microarrays; juvenile and ongrowing intestine/liver qPCR.	Custom Agilent 8×60K one-color microarray (GPL15840); RT-qPCR validation.	Forty-eight individual microarrays; qPCR used separate individual fish at juvenile and ongrowing stages.	Scale/depth: NA for read depth—microarray platform. Each array received 600 ng Cy3-labeled cRNA. QC/mapping: Juvenile qPCR samples RIN ≥ 9. Microarray acceptance required cRNA yield > 0.825 µg and specific activity > 6 pmol Cy3/µg; scanned at 3-µm resolution.	One-way ANOVA $p < 0.05$; article figures/gene lists also refer to FDR 0.05. GO enrichment used Benjamini–Hochberg-corrected EASE score < 0.05.	GEO GSE84985; platform GPL15840.	[14]

Species	Tissue and molecular endpoint	Platform and analytical workflow	Library construction or pooling	Sequencing scale, quality, and mapping	Molecular or statistical criterion	Public data accession or reporting status	References
Gilthead sea bream (*Sparus aurata*)	Broodstock blood *fads2*; offspring liver lipid-metabolism genes; *fads2* promoter methylation.	RT-qPCR for parental screening; Bio-Rad QX200 droplet digital PCR for offspring genes; PyroMark Q24 pyrosequencing.	Targeted qPCR/ddPCR/pyrosequencing; small methylation subsets and some pooled liver samples.	Scale/depth: NA—targeted qPCR/ddPCR and pyrosequencing study. QC/mapping: ddPCR readings with < 12,000 droplets were excluded; methylation runs had to pass PyroMark quality control.	Two-way ANOVA with Tukey comparisons at $p < 0.05$; Pearson correlations were used for methylation/expression relationships.	No high-throughput sequencing accession applicable.	[15]
Golden pompano (*Trachinotus ovatus*)	Muscle transcriptome and qPCR of glycerolipid/phospholipid acyltransferases.	Illumina NovaSeq 6000 RNA-seq; de novo Trinity assembly; CFX96 qPCR validation.	qPCR retained cage-level replication; the RNA-seq library number and pooling structure were not clearly reported.	Scale/depth: 43,631,590 clean reads for D2 and 45,273,460 for D5; the article reports group-level totals rather than clearly resolved per-library depth. QC/mapping: Reads with adapters, > 10% ambiguous bases, or > 50% bases with Q ≤ 20 were removed. The article prints a mean mapped-read value of “1844,” but the metric/unit is unclear.	The main article does not state a numerical DEG fold-change/FDR threshold. qPCR and lipid outcomes used one-way ANOVA with Tukey’s test, $p < 0.05$.	No public accession stated in the reviewed article; library replication and mapping reporting were unclear.	[16]
Common carp (*Cyprinus carpio*)	Intestine; antioxidant, inflammatory, tight-junction, and villification genes plus intestinal-content microbiota.	Targeted qPCR and Illumina 16S rRNA amplicon sequencing (V3–V4).	Three pooled 16S samples/treatment; each pool was derived from one tank.	Scale/depth: NR; the article does not report reads/sample or total sequence yield. QC/mapping: OTUs clustered at 97% identity; taxonomy assigned using the SILVA database with 70% confidence. qPCR reactions were technical triplicates.	One-way ANOVA followed by Tukey’s test; $p < 0.05$.	SRA SRR12130498-SRR12130512.	[26]
Gilthead sea bream (*Sparus aurata*)	Liver, head kidney, and posterior intestine PCR arrays; posterior-intestinal adherent microbiota.	Customized 96-well qPCR arrays; Illumina MiSeq 16S V3–V4, 2×300 bp.	Eighteen individual paired host-gene/microbiota samples; no pooling reported.	Scale/depth: 4,068,405 high-quality merged reads across 18 samples; mean 226,022 reads/sample. QC/mapping: Reads < 150 bp, terminal Phred < 28, or mean quality < 26 were discarded; 1,712 OTUs at 97% identity. qPCR efficiencies > 92% and $r^2 > 0.98$.	Gene expression: t tests/ANOVA at $p < 0.05$. Microbiota: Kruskal–Wallis/Dunn $p < 0.05$; PERMANOVA with 10,000 permutations; OTU–gene correlations $p < 0.001$.	SRA PRJNA750446; BioSamples SAMN20458383-SAMN20458400.	[24]

Species	Tissue and molecular endpoint	Platform and analytical workflow	Library construction or pooling	Sequencing scale, quality, and mapping	Molecular or statistical criterion	Public data accession or reporting status	Reference s
Gilthead sea bream (*Sparus aurata*)	Anterior-intestinal adherent microbiota across the production cycle; anterior-intestinal RNA-seq at the intermediate sampling.	Illumina MiSeq 16S V3–V4, 2×300 bp; Illumina HiSeq 2500 RNA-seq, 2×150 bp; DESeq2.	Ninety-four individual 16S samples across time; 36 individual RNA-seq libraries at the intermediate stage.	Scale/depth: 16S: approximately 10 million high-quality reads total. RNA-seq: approximately 1,000 million paired-end reads total, averaging ~28 million/sample; 21–34 million retained reads/sample after trimming. QC/mapping: RNA RIN 8–10; approximately 1% RNA reads discarded during trimming; mapping reached up to 93%. 16S yielded 1,892 OTUs and an observed/expected OTU ratio near 70%.	RNA-seq DESeq2 FDR < 0.05. Microbiota PERMANOVA used 10,000 permutations; pathway and inferred-function testing applied FDR control.	BioProject PRJNA876784; DNA BioSamples SAMN30672395-SAMN30672488; RNA BioSamples SAMN30672489-SAMN30672524.	[25]
Cobia (*Rachycentron canadum*)	Intestine, liver, and dorsal muscle amino-acid transport and TOR/IGF signaling; dorsal-muscle phosphoproteins.	SYBR RT-qPCR; Western blot for phosphorylated TOR, S6K, and 4E-BP1.	Targeted qPCR and Western blot on tissue pools formed within cages.	Scale/depth: NA—targeted qPCR and protein assay. QC/mapping: *β-actin* reference; 2−ΔΔCt; Western blot used target-specific phospho-antibodies.	One-way ANOVA with post hoc mean comparison at $p < 0.05$; broken-line regression estimated methionine requirement.	No sequencing accession applicable.	[30]
Largemouth bass (*Micropterus salmoides*)	Liver, intestine, and muscle nutrient-sensing genes; intestinal and muscle peptide/amino-acid transporters; liver and muscle signaling proteins.	SYBR RT-qPCR and Western blot/phosphoprotein analysis.	Targeted qPCR and Western blot on tissue pools formed within tanks.	Scale/depth: NA—targeted transcript and protein assays. QC/mapping: Primer efficiencies were checked using serial cDNA dilutions; 2−ΔΔCt normalization; protein phosphorylation provided orthogonal validation.	Independent-samples t test for two diets; $p < 0.05$.	No sequencing accession applicable.	[31]
Senegalese sole (*Solea senegalensis*)	Liver; bile-acid and lipid-metabolism genes including *cyp7a1*, *taut*, *abcc2*, *abcb11*, apolipoproteins, *mtp*, *vldlr*, and *plin2*.	StepOnePlus RT-qPCR with SsoFast EvaGreen; 2−ΔΔCt; log2-transformed expression.	Targeted qPCR using individual liver samples distributed across replicate tanks.	Scale/depth: NA—targeted qPCR study. QC/mapping: RNA purity by NanoDrop and integrity by agarose gel; gene-specific PCR efficiencies verified near 100%; melting curves and minus-RT controls; *gapdh* stability tested with RefFinder.	One-way ANOVA or nonparametric alternatives as appropriate, with Tukey HSD/pairwise tests; $p < 0.05$.	Data available from the authors on request; no sequencing accession applicable.	[32]

Species	Tissue and molecular endpoint	Platform and analytical workflow	Library construction or pooling	Sequencing scale, quality, and mapping	Molecular or statistical criterion	Public data accession or reporting status	References
Silver sillago (*Sillago sihama*)	Liver and intestine; *Nrf2*, *Keap1*, *GST*, *GPx*, *CAT*, *CuZnSOD*, *MnSOD*, and *GR* expression.	ABI 7500 SYBR RT-qPCR; *GAPDH* normalization; 2−ΔΔCt.	Targeted qPCR using fish sampled from each replicate tank.	Scale/depth: NA—targeted qPCR study. QC/mapping: RNA extracted with TRIzol and reverse-transcribed with M-MLV; GAPDH stability was supported by prior transcriptomic work; primer sequences and amplicon sizes were reported.	One-way ANOVA followed by Duncan's multiple-range test, $p < 0.05$; polynomial contrasts and regression models were used for requirement estimates.	No sequencing accession applicable.	[34]
Olive flounder (*Paralichthys olivaceus*)	Brain and liver; *hsp60*, *hsp70*, *hsp90*, and *wap65* after handling or acute thermal exposure.	StepOne RT-qPCR with TB Green; *β-actin* normalization; 2−ΔΔCt.	Targeted qPCR with tank-level replication; blood was pooled by tank.	Scale/depth: NA—targeted qPCR study. QC/mapping: RNA A260/A280 2.21 ± 0.01; melting-curve verification. Primer efficiencies 96.8–100.1%, with reported r^2 and slopes.	Two-way ANOVA for diet × temperature, Type III sums of squares; Tukey HSD at $p < 0.05$. Dose-response regressions were also evaluated.	No sequencing accession applicable.	[33]
Tilapia (*Oreochromis* spp.)	Liver and muscle transcriptomes/qPCR; full-length intestinal 16S rRNA microbiome.	Short-read RNA-seq aligned with HISAT2/StringTie; qRT-PCR; Oxford Nanopore MinION/Flongle full-length V1–V9 16S sequencing.	Transcriptome library construction was unclear relative to 15 sampled fish and 6 deposited runs; microbiome n = 3/group.	Scale/depth: RNA-seq read length and reads/library NR. Nanopore sequence yield/read count NR. QC/mapping: RNA reads adapter/quality trimmed with TrimGalore and aligned to GCF_000188235.2. Nanopore reads retained at 1450–1650 bp with Q ≥ 7 and aligned using Minimap2.	DE selection: FPKM fold change ≥ 2 and summed expression ≥ 1; enrichment two-sided hypergeometric $p \leq 0.05$. Other outcomes used one-way ANOVA/Tukey $p < 0.05$; microbiome PERMANOVA and t tests.	RNA BioProject PRJNA889219; SRA SRR21869238-SRR21869243; microbiome accession not stated.	[39]
Hybrid grouper (*E. fuscoguttatus* ♀ × *E. lanceolatus* ♂)	Liver, muscle, spleen, and head-kidney qPCR; intestinal microbiota.	LightCycler 480 qPCR; Illumina MiSeq 16S V4–V5 amplicon sequencing; QIIME/UPARSE.	Targeted qPCR reported n = 4 replicates, but pooling or within-tank handling was not described; microbiota sampled from 4 fish/group.	Scale/depth: NR; reads/sample and total sequence yield were not reported. QC/mapping: RNA concentration/purity/integrity assessed by microplate photometry and agarose gel. qPCR efficiencies 94–105%. 16S OTUs clustered at 97% identity after Trimmomatic/FLASH processing.	One-way ANOVA and Tukey HSD; $p < 0.05$. Pearson correlations linked taxa with host outcomes.	No sequencing accession reported.	[40]

Species	Tissue and molecular endpoint	Platform and analytical workflow	Library construction or pooling	Sequencing scale, quality, and mapping	Molecular or statistical criterion	Public data accession or reporting status	References
Grey mullet (*Mugil cephalus*)	Liver and spleen immune-gene qPCR; intestinal microbiota in control and *L. rhamnosus* groups.	MyiQ RT-qPCR; Illumina HiSeq 2000 16S V3–V4, 2×250 bp; QIIME2/UPARSE/LEfSe.	Targeted qPCR and 16S from fish sharing one tank/group; microbiome restricted to control and *L. rhamnosus*.	Scale/depth: NR; the article does not report total reads or reads/sample. QC/mapping: 16S OTUs constructed at 97% identity; chimeras removed; *β-actin* used for qPCR normalization. LEfSe threshold LDA score 2.	One-way ANOVA with Tukey HSD, $p < 0.05$; microbiome PERMANOVA; Kaplan–Meier/log-rank survival; Spearman correlations.	Microbiome OTU data were supplied in supplementary information; no public accession was stated.	[41]

Footnote: Biological sample numbers and experimental units are reported in Table 1 and are not repeated here except where pooling or library construction is necessary to interpret the molecular analysis. Molecular-response magnitudes are reported in Table S1. NR indicates not reported in the reviewed article; NA, not applicable. No fold changes or sequencing metrics were estimated from figures. qPCR performed on the same RNA used for sequencing was treated as cross-platform confirmation rather than independent biological validation.

Abbreviations: 4E-BP1, eukaryotic translation initiation factor 4E-binding protein 1; 16S rRNA, 16S ribosomal RNA; bp, base pair; cDNA, complementary DNA; ddPCR, droplet digital polymerase chain reaction; DEG, differentially expressed gene; FDR, false discovery rate; FM, fishmeal; FPKM, fragments per kilobase per million; GEO, Gene Expression Omnibus; LDA, linear discriminant analysis; LC-MS, liquid chromatography-mass spectrometry; miRNA, microRNA; NA, not applicable; NR, not reported; OTU, operational taxonomic unit; qPCR, quantitative polymerase chain reaction; RIN, RNA integrity number; RNA-seq, RNA sequencing; SBM, soybean meal; SRA, Sequence Read Archive; VIP, variable importance in projection.
